# Mussel‐Bioinspired Edible Ca^2+^‐Crosslinked Alginate Hydrogel Electrodes for Glucose Gastrointestinal Monitoring

**DOI:** 10.1002/advs.202516912

**Published:** 2025-12-02

**Authors:** Verdiana Marchianò, Claudio Pellegrini, Angelo Tricase, Eleonora Macchia, Andrea Brattelli, Luigi Gentile, Patrizia Nadia Hanieh, Noemi Fiaschini, Antonio Rinaldi, Luisa Torsi, Paolo Bollella

**Affiliations:** ^1^ Department of Chemistry University of Bari Aldo Moro Via E. Orabona 4 Bari 70125 Italy; ^2^ Centre for Colloid and Surface Science (CSGI@UniBa) University of Bari Aldo Moro Via E. Orabona 4 Bari 70125 Italy; ^3^ Department of Pharmacy‐Pharmaceutical Sciences University of Bari Aldo Moro Via E. Orabona 4 Bari 70125 Italy; ^4^ Faculty of Science and Engineering Åbo Akademi University Turku 20500 Finland; ^5^ Aerospace Sciences and Engineering (Inter‐University Ph.D.) Polytechnic of Bari University of Bari Aldo Moro Via Orabona 4 Bari 70126 Italy; ^6^ Nanofaber S.r.l Via Anguillarese 301 Rome 00123 Italy; ^7^ Department of Sustainability Circularity and Climate Change Adaptation of Production and Territorial Systems (SSPT) Research Centre of Casaccia, ENEA Via Anguillarese 301, Santa Maria di Galeria Rome 00123 Italy; ^8^ Department of Energy Technologies and Renewable Sources (TERIN) Research Centre of Casaccia, ENEA Via Anguillarese 301, Santa Maria di Galeria Rome 00123 Italy

**Keywords:** Ca^2+^‐crosslinked alginate, conducting hydrogels, edible electrodes, glucose intestinal sensing, polydopamine

## Abstract

A novel self‐standing, edible polydopamine‐based alginate‐hydrogel electrode that intrinsically conducts ions and electrons is introduced, redefining the architecture of ingestible bioelectronics. The edible polydopamine‐based alginate‐hydrogel electrode are based on Ca^2^⁺‐crosslinked alginate (3.5% w/v) plasticized with glycerol (5% w/v) and reinforced with polydopamine, silver nanoparticles, and food‐grade glucose oxidase. The optimized formulation exhibits an electroactive surface area of 1.99 ± 0.07 cm^2^, a double‐layer capacitance of 10.1 ± 0.3 µF, and a charge‐transfer resistance of 7.7 ± 0.6 kΩ. Structural characterization by SEM, TEM, AFM, WAXS, and FTIR confirms uniform dispersion of AgNPs, pDA domain formation, and stable enzyme incorporation, while rheology and DMA reveal enhanced viscoelasticity, tensile strength (14 MPa), and Young's modulus (65 MPa). Configured as a first‐generation glucose biosensor operating in USP simulated intestinal fluid (pH 6.8), the electrode displays a linear response from 50 µm to 1.0 mm, a detection limit of 10.4 ± 0.8 µm, and an apparent K_M_^app^ of 0.35 ± 0.08 mm. The biosensor retains ≥95% activity during 20 h of continuous operation and 90% after 30 days storage, with negligible interference from physiological species. This edible platform establishes a robust route toward ingestible bioelectronics for non‐invasive glucose monitoring and personalized metabolic management.

## Graphical abstract

A fully edible soft electrode is engineered from Ca^2^⁺‐crosslinked alginate integrated with polydopamine, silver nanoparticles, and food‐grade glucose oxidase. The hybrid hydrogel combines ionic and electronic conduction, enabling efficient glucose‐to‐H_2_O_2_ conversion and catalytic reduction in simulated intestinal fluid. This strategy demonstrates a sustainable pathway toward ingestible electrochemical biosensors for real‐time metabolic monitoring.

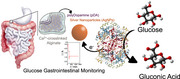

## 1 Introduction

The integration of soft matter with bioelectronic technologies is redefining how electronic systems interface with living tissues.^[^ ^1^, ^2^, ^3^, ^4^ ^]^ Within this landscape, hydrogel‐based electrodes are emerging as highly promising candidates for next‐generation biointerfaces due to their intrinsic softness, high water content, and excellent biocompatibility.^[^ ^5^, ^6^, ^7^ ^]^ In contrast, conventional metallic electrodes, while electronically conductive and chemically stable, exhibit several fundamental drawbacks.^[^ ^8^ ^]^ Their rigid mechanical properties produce a mismatch with compliant biological tissues, frequently resulting in chronic inflammation and fibrotic encapsulation.^[^ ^9^, ^10^ ^]^ Moreover, their electrochemical performance is intrinsically limited: metallic electrodes primarily operate via double‐layer capacitance, which restricts charge injection capacity, and they lack reversible redox activity in biologically relevant potential windows.^[^ ^11^ ^]^ Consequently, they cannot efficiently support bioelectrocatalytic processes or dynamic signal amplification.^[^ ^12^ ^]^ Additional limitations include chemically inert surfaces that hinder the direct immobilization of bioactive species and susceptibility to fouling phenomena, which compromise stability during long‐term use.^[^ ^13^ ^]^ These challenges highlight the need for alternative electrode materials that are soft, redox‐active, and intrinsically biocompatible, while also supporting efficient and stable charge transfer under physiological conditions.^[^ ^14^, ^15^, ^16^ ^]^

Conducting hydrogels (CHs) address these challenges by combining the electrical functionality of conducting polymers (CPs) with the mechanical compliance of hydrated networks.^[^ ^17^, ^18^, ^19^, ^20^ ^]^ Embedding CPs within polymeric hydrogels alleviates the brittleness and poor biofunctional integration capacity of CPs, while preventing the dehydration and fragility often observed in pristine hydrogels.^[^ ^21^ ^]^ The resulting hybrid structures, frequently organized as interpenetrating polymer networks (IPNs), provide robust electron transport pathways, improved charge storage capability, and compatibility with embedded biomolecules.^[^ ^22^ ^]^ These multifunctional composites have already enabled progress in diverse fields, ranging from neural stimulation and electrophysiological monitoring to electro‐responsive drug delivery and biosensing.^[^ ^23^, ^24^, ^25^, ^26^ ^]^ Despite these advances, the design of edible, biodegradable, and physiologically compatible CHs remains an underexplored domain, one with significant potential for transient bioelectronics and gastrointestinal (GI) monitoring.^[^ ^27^ ^]^

Moreover, calcium‐crosslinked alginate hydrogels offer a versatile and biocompatible matrix. Ionic gelation with Ca^2^⁺ ions yields soft, mechanically stable, and biodegradable scaffolds, which can encapsulate delicate biomolecules under mild conditions.^[^ ^28^, ^29^ ^]^ Functionalization with polydopamine (pDA), inspired by mussel adhesion chemistry, introduces electronic conductivity, reactive surface groups, and intrinsic redox activity, thereby transforming the alginate hydrogel into a conductive nanohybrid system.^[^ ^30^, ^31^, ^32^ ^]^ In addition to enhancing electron transport, PDA provides bioadhesion and chemical versatility, facilitating the immobilization of enzymes, mediators, and nanomaterials required for bioelectrocatalysis.^[^ ^33^, ^34^ ^]^ The combination of Ca^2^⁺‐crosslinked alginate with PDA thus defines a soft, edible, and electroactive platform capable of stable performance in the dynamic and chemically challenging environment of the GI tract.^[^ ^35^, ^36^ ^]^

Further functionalization is achieved through the entrapment of redox enzymes within the hydrogel architecture.^[^ ^37^, ^38^ ^]^ Biocatalysts such as glucose oxidase (GOx) and pyrroloquinoline quinone–dependent glucose dehydrogenase (PQQ‐GDH) maintain their native structure and activity when confined within hydrated polymer networks.^[^ ^39^, ^40^ ^]^ The porous nature of alginate–pDA hydrogels ensures rapid diffusion of substrates and reaction products, while the quinone/hydroquinone groups of pDA participate in redox mediation, promoting efficient electron transfer.^[^ ^41^ ^]^ This integrated configuration couples enzymatic specificity with electronic transduction, enabling the fabrication of bioelectrodes that are simultaneously soft, selective, and conductive.^[^ ^36^ ^]^

The clinical motivation for such systems is particularly compelling in the field of gastrointestinal glucose monitoring, an area of growing relevance for the management of type 2 diabetes (T2D) and related metabolic disorders.^[^ ^42^, ^43^, ^44^ ^]^ With the global prevalence of metabolic disease continuing to increase, postprandial glucose absorption in the small intestine has become a critical therapeutic target.^[^ ^45^ ^]^ Existing monitoring technologies are invasive or fail to capture local fluctuations in glucose during digestion, limiting their clinical utility.^[^ ^46^, ^47^ ^]^ Ingestible biocatalytic sensors could address this gap by enabling real‐time, site‐specific, and transient monitoring of glucose levels within the GI tract, while simultaneously interfacing with controlled drug release platforms.^[^ ^48^, ^49^ ^]^ Such devices could be integrated into capsules capable of releasing sodium–glucose cotransporter 1 (SGLT1) inhibitors or other glucose‐modulating agents in response to detected spikes, transmitting data wirelessly to external devices and thereby enabling adaptive, personalized glycemic management.^[^ ^36^ ^]^

However, the practical deployment of ingestible devices remains hampered by the reliance on non‐degradable materials, the risk of device retention, and the requirement for medical supervision or retrieval.^[^ ^50^ ^]^ The emergence of edible electronics, developed based on green chemistry and biodegradable building blocks, provides a paradigm shift in this context. These systems are designed to be safely ingested, to perform transiently, and to undergo complete degradation after use. By eliminating retrieval procedures and aligning with environmentally sustainable principles, edible bioelectronics open a new avenue for transient devices that extend far beyond healthcare, with transformative implications for personalized medicine and sustainable technology.^[^ ^51^ ^]^

This work establishes a new materials paradigm for ingestible bioelectronics by introducing a self‐standing, fully edible, and intrinsically conductive hydrogel electrode that operates without any current collector. Unlike prior alginate, or pDA‐based hydrogel coatings, the Ca^2^⁺–alginate/polydopamine/AgNPs matrix here forms a cohesive ionic–electronic percolation network that directly performs electrochemical transduction, revealing unprecedented mixed conduction in an edible format and enabling the first quantitative glucose sensing under simulated intestinal conditions.

A comprehensive multiscale characterization is undertaken to evaluate the electrochemical, structural, and mechanical properties of the system. Cyclic voltammetry (CV), amperometry, and electrochemical impedance spectroscopy (EIS) are employed to probe charge transfer dynamics, catalytic activity, and ionic diffusion within the conductive hydrogel network. Wide‐Angle X‐ray Scattering (WAXS) is applied to investigate molecular and crystalline ordering, while rheological and dynamic mechanical analysis (DMA) are used to assess viscoelastic and mechanical performance. Morphological and structural features are further examined by scanning electron microscopy (SEM), transmission electron microscopy (TEM), and atomic force microscopy (AFM), in combination with Fourier‐transform infrared spectroscopy (FTIR) to identify molecular interactions among the functional components.

The results of this investigation demonstrate that the developed pDA–alginate hydrogel electrode successfully combines mechanical robustness, electrochemical conductivity, and biological compatibility. The system represents a promising platform for the realization of ingestible enzymatic biosensors intended for non‐invasive glucose monitoring in the gastrointestinal tract, thereby advancing the field of edible bioelectronics.

## 2 Results and Discussion

### 2.1 Electrochemical Characterization of pDA‐Ca^2+^‐Crosslinked Alginate Hydrogel Electrodes

**Figure 1:**
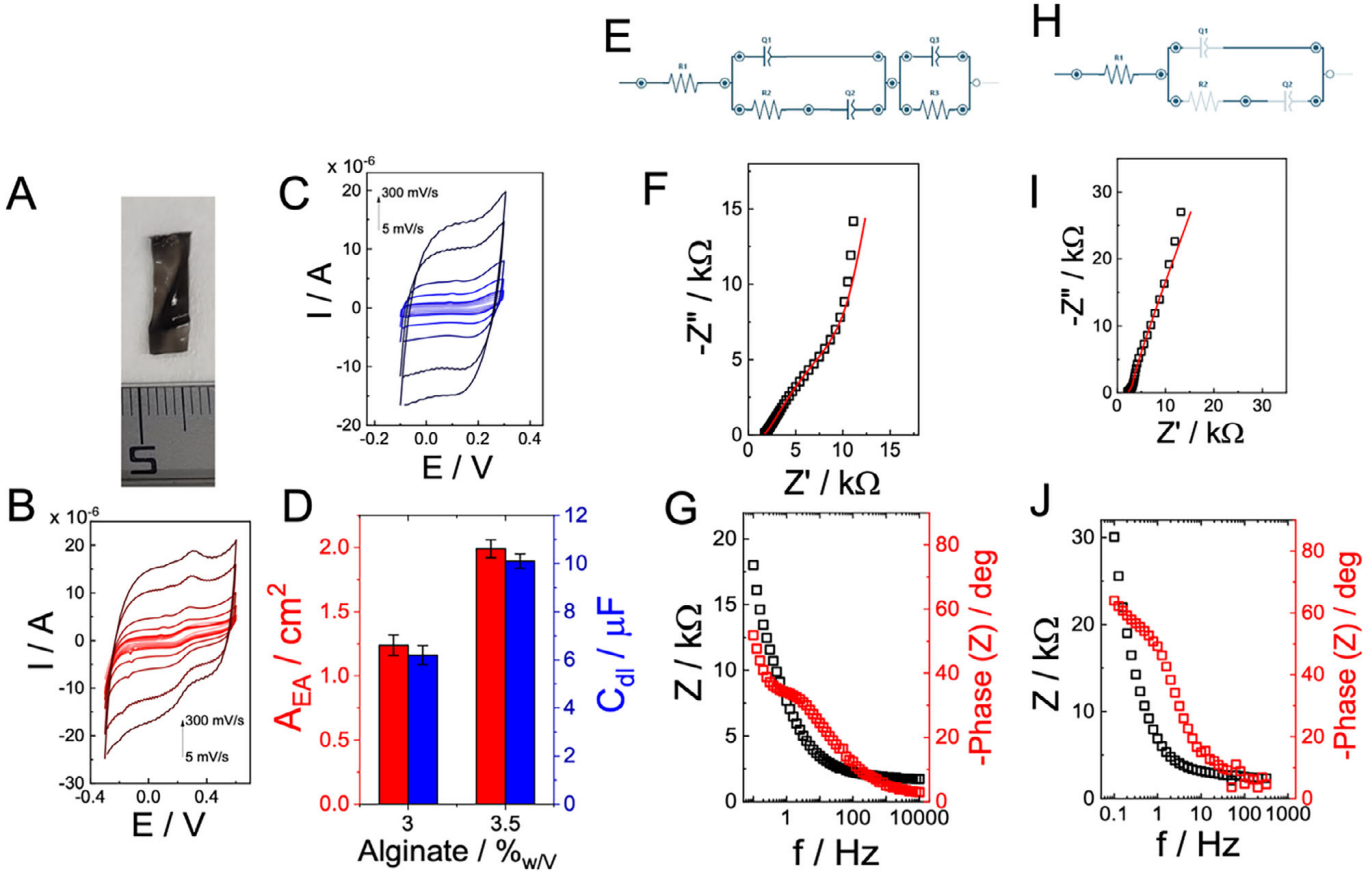
A) Representative photograph of a free‐standing Ca^2^⁺‐crosslinked alginate–pDA hydrogel electrode (3.5% w/v). B) Cyclic voltammograms (CVs) recorded in 10 mm HEPES buffer (pH 7.2, 100 mm KCl) containing 10 mm [Fe(CN)₆]^3−^/^4−^ for electrodes prepared with 3.5% (w/v) alginate, at scan rates ranging from 5 to 300 mV s^−1^. C) CVs of the same electrodes in blank supporting electrolyte (HEPES 10 mm, pH 7.2, 100 mm KCl) in the absence of redox probe, used to extract capacitive currents and estimate double‐layer capacitance. D) Electroactive area (A_EA_, red) and double‐layer capacitance (C_dl_, blue) determined for electrodes containing 3.0% and 3.5% (w/v) alginate (common reference formulation considered as baseline for comparisons: 3.5% w/v alginate, 5% w/v glycerol, 1 mL pDA per batch). E–J) Electrochemical impedance spectroscopy (EIS) analysis. Equivalent circuit models employed for fitting are reported in panels (E,H). F,G) Nyquist and Bode plots, respectively, for 3.0% w/v alginate electrodes, fitted with the circuit shown in (E). H,J) Nyquist and Bode plots, respectively, for 3.5% w/v alginate electrodes, fitted with the circuit shown in (I). Experimental data are shown as black squares, and red lines correspond to fits.

**Figure** **1**A shows the free‐standing pDA–alginate hydrogel electrode prepared by Ca^2^⁺ crosslinking of alginate in the presence of polydopamine. The electrode exhibits structural integrity sufficient for direct manipulation and mounting in the electrochemical cell, confirming that the hydrogel can function as a self‐supporting soft electrode without the need for rigid backing materials.

The electroactivity was first assessed in the presence of 10 mm [Fe(CN)₆]^3−/4−^ with 100 mM KCl and 10 mM HEPES (pH 7.2). Figure 1B shows cyclic voltammograms recorded at scan rates ranging from 5 to 300 mV s^−1^.^[^ ^52^ ^]^ Both anodic and cathodic peaks are visible, with peak currents (I_p_) scaling with the square root of scan rate (ν^1/2^). This behavior indicates that electron transfer at the hydrogel/electrolyte interface is governed by semi‐infinite diffusion of the redox probe, in agreement with the Randles–Ševčík relation (Equation 1):

(1) $$I_{p}=\left(2.69\times 10^{5}\right)\cdot n^{3/2}\cdot A\cdot D^{1/2}\cdot C\cdot \nu^{1/2}$$

where *n* = 1, A is the electroactive area, D is the diffusion coefficient of [Fe(CN)₆]^3−/4−^ (7.6 × 10^−6^ cm^2^ s^−1^), C is the concentration, and ν is the scan rate. By applying this model, the electroactive area (A_EA_) was determined as 1.24 ± 0.08 cm^2^ for hydrogels containing 3.0% alginate and 1.99 ± 0.07 cm^2^ for hydrogels with 3.5% w/v alginate (Figure 1D). The higher A_EA_ at 3.5% w/v alginate indicates that crosslinking density influences the electronic percolation pathways within the pDA‐containing matrix.

To evaluate intrinsic capacitive behavior, CVs were recorded in supporting electrolyte (10 mm HEPES, 100 mm KCl, pH 7.2) without redox species.^[^ ^53^ ^]^ The resulting voltammograms, shown in Figure 1C, exhibit rectangular profiles, characteristic of non‐faradaic double‐layer charging. The capacitive current was extracted at different scan rates and plotted versus scan rate, with the slope yielding the double‐layer capacitance (C_dl_). The calculated values were 6.2 ± 0.4 µF for 3.0% w/v alginate and 10.1 ± 0.3 µF for 3.5% w/v alginate (Figure 1D). The increase in capacitance with higher alginate concentration reflects enhanced ionic accessibility and a larger effective double‐layer region within the hydrated matrix.

The impedance response of pDA–alginate hydrogel electrodes was analyzed using equivalent circuit modelling to investigate the mixed ionic‐electronic conduction mechanism. For the 3.0% w/v alginate hydrogel, the experimental Nyquist and Bode plots (Figure 1G,H) were fitted with the circuit shown in Figure 1E.^[^ ^54^, ^55^ ^]^ The model comprises the series solution resistance (R_S_), a constant phase element (CPE_1_), and two parallel branches: one formed by R_CT_–CPE_2_ and the second by R_CT2_–CPE_3_. The impedance of each constant phase element is defined as Equation (2):

(2) $$Z_{CPE}=\frac{1}{Q\cdot {\left(j\omega \right)}^{n}}$$

where Q is the pseudo capacitance parameter, ω the angular frequency, and *n* (0 ≤ *n* ≤ 1) describes deviation from ideal capacitive behavior.^[^ ^56^ ^]^ The total impedance of the circuit is Equation (3):

(3) $$Z\left(\omega \right)=R_{s}+\left[\frac{1}{\frac{1}{Z_{CPE1}}+\frac{1}{\;R_{CT}+Z_{CPE2}}}\right]+\left[\frac{1}{\frac{1}{Z_{CPE3}}+\frac{1}{R_{CT2}}}\right]$$

The fitting parameters were R_S_ = 1597 ± 64 Ω, R_CT_ = 22,750 ± 2958 Ω, R_CT2_ = 2618 ± 212 Ω, CPE_1_ = 38 ± 2 µF (*n* = 0.8 ± 0.1), CPE_2_ = 49 ± 2 µF (*n* = 1.0 ± 0.1), and CPE_3_ = 186 ± 19 µF (*n* = 0.9 ± 0.1), with χ^2^ = 0.0002 (Table S1, Supporting Information). The Nyquist spectrum (Figure 1G) shows two semicircles, consistent with the presence of two distinct charge‐transfer processes, followed by a diffusion‐related tail at low frequencies. The corresponding Bode plot (Figure 1H) reveals a phase angle approaching –80° at low frequencies, indicative of capacitive control, and a shift toward –45° in the 10–100 Hz region, reflecting the interplay between diffusion within the hydrogel matrix and charge transfer through pDA domains.^[^ ^57^ ^]^

For the 3.5% w/v alginate hydrogel, the impedance spectra (Figure 1I,J) were fitted using the equivalent circuit in Figure 1H. The circuit consists of R_S_ in series with CPE_1_, followed by a single R_CT_–CPE_2_ branch. The total impedance is:

(4) $$Z\left(\omega \right)=R_{s}+\left[\frac{1}{\frac{1}{Z_{CPE1}}+\frac{1}{\;R_{CT}+Z_{CPE2}}}\right]$$

Fitting yielded R_S_ = 2270 ± 74 Ω, R_CT_ = 3779 ± 261 Ω, CPE_1_ = 41 ± 1 µF (*n* = 0.9 ± 0.1), and CPE_2_ = 7 ± 1 µF (*n* = 0.8 ± 0.1), with χ^2^ = 0.0014 (Table S1, Supporting Information). The Nyquist spectrum (Figure 1I) shows a single semicircle with a lower diameter compared to the 3.0% w/v hydrogel, consistent with a reduced charge‐transfer resistance.^[^ ^58^, ^59^ ^]^ The Bode plot (Figure 1J) again shows capacitive dominance at low frequencies with a phase angle near –80°, but with less pronounced resistive character in the mid‐frequency region, consistent with the lower R_CT_.

The pDA–alginate hydrogel electrodes operate as mixed ionic–electronic interfaces in which bulk ionic transport through a hydrated network couples to electron transfer at pDA‐rich domains; accordingly, we model the spectra with simplified, physically grounded elements routinely used for conductive hydrogels. In both formulations, the series solution/bulk term R_S_ captures through‐gel/electrolyte ionic resistance set by water content and Ca^2^⁺ “egg‐box” crosslinking. The non‐ideal interfacial term CPE_1_ describes the distributed double layer that forms where hydrated ions meet the rough/soft hydrogel–electrode contact; its exponent *n* < 1 reflects time‐constant dispersion typical for hydrated porous media. These assignments (bulk R for ionic transport, CPE for interfacial charge storage at blocking electrodes) follow the standardized hydrogel EIS framework in which ionic and electronic contributions are resolved with minimal circuit complexity.^[^ ^60^ ^]^ For the 3.0% w/v alginate gel, the Nyquist/Bode plots (require two parallel kinetic channels in series after R_S_–CPE_1_, implemented as (R_CT_∥CPE_2_) and (R_CT2_∥CPE_3_), quantitatively associated with a slow, poorly coupled route (R_CT_ = 22.8 ± 3.0 kΩ) that runs in parallel with a faster channel (R_CT2_ = 2.62 ± 0.21 kΩ). The former can be attributed to less‐connected hydrogel regions that hinder ion–electron coupling, and the latter to pDA‐rich microdomains that support redox‐hopping once hydrated ions access them. The associated CPEs represent non‐ideal local double‐layer/charging behavior of each route. This lumped two‐branch description is the compact analogue of the parallel ionic/electronic pathways emphasized for conductive hydrogels, where separable processes appear as distinct RC features when time constants are resolved.^[^ ^61^ ^]^

For the 3.5% w/v alginate gel, increased crosslink density suppresses interfacial heterogeneity and the response collapses to a single kinetic branch, R_S_–CPE_1_–(R_CT_∥CPE_2_), with a six‐fold decrease in charge‐transfer resistance, indicating a more uniform double layer and improved overlap between ionic pathways and pDA redox sites. The rise in R_S_ from 1597 to 2270 Ω is consistent with a denser Ca^2+^‐alginate network slightly reducing bulk ionic mobility, but the kinetics are dominated by the large drop in R_CT_, yielding a smaller Nyquist diameter and less resistive mid‐frequency phase response.^[^ ^62^ ^]^

Comparison of the two formulations indicates that the 3.0% w/v alginate hydrogel electrode requires a more complex model with two distinct charge‐transfer processes and multiple CPEs, while the 3.5% w/v alginate hydrogel electrode can be described by a simpler circuit with a single R_CT_. The charge‐transfer resistance decreases from ≈22.8 kΩ (3.0%) to ≈3.8 kΩ (3.5%), demonstrating that higher crosslinking density improves ion–electron coupling and reduces interfacial heterogeneity.^[^ ^63^ ^]^

### 2.2 Optimization of Glycerol and pDA Within pDA‐Ca^2+^‐Crosslinked Alginate Hydrogel Electrodes

**Figure 2:**
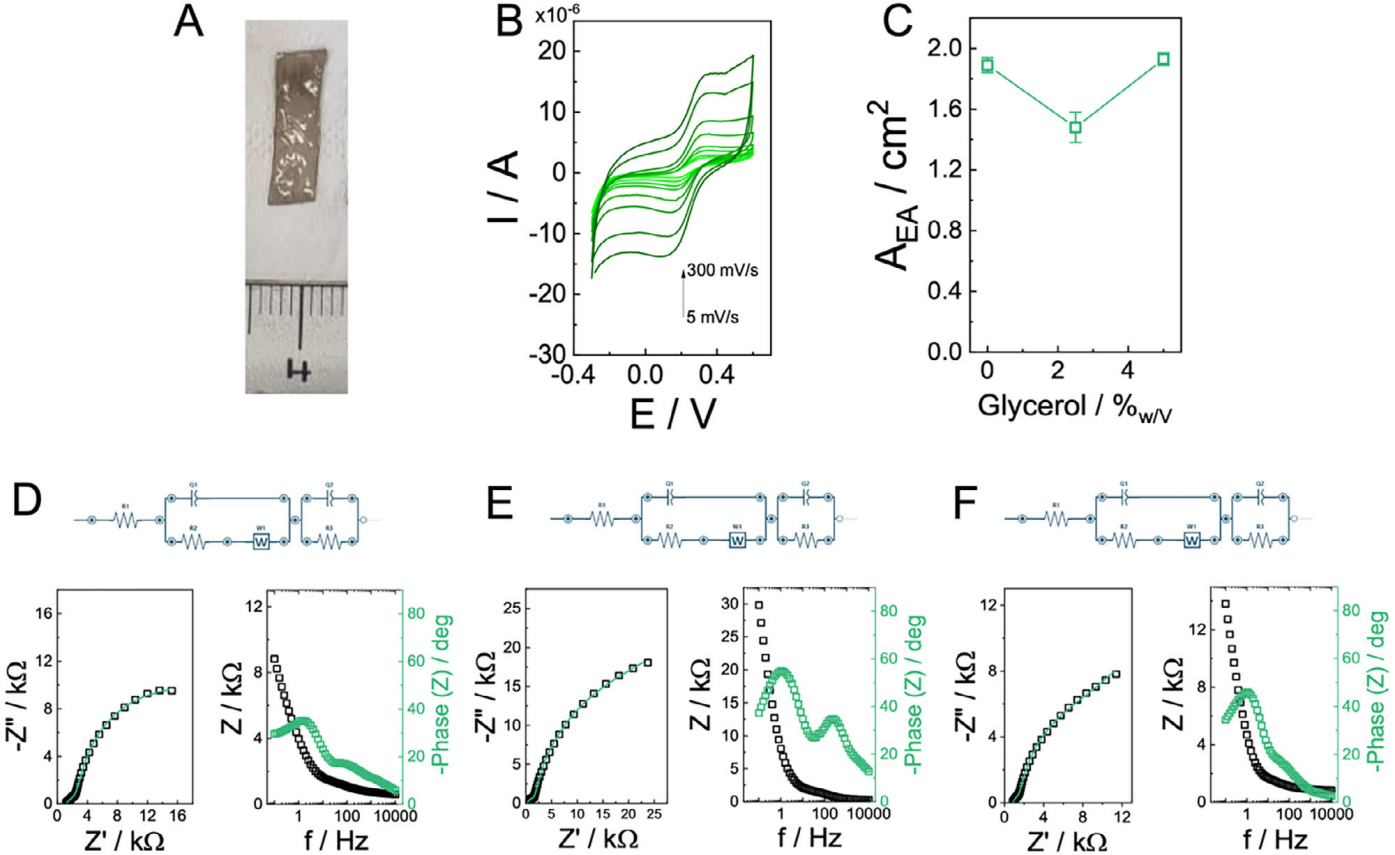
A) Representative photograph of a free‐standing hydrogel electrode prepared with 3.5% (w/v) alginate, 1 mL pDA, and 5% w/v glycerol content. B) Cyclic voltammograms (CVs) recorded in 10 mm HEPES buffer (pH 7.2, 100 mm KCl) containing 10 mm [Fe(CN)₆]^3−^/^4−^ for electrodes incorporating different glycerol contents, at scan rates ranging from 5 to 300 mV s^−1^. C) Electroactive area (A_EA_) as a function of glycerol content (0, 2.5, and 5% w/v), calculated from CVs using the Randles–Ševčík equation. D–F) Electrochemical impedance spectroscopy (EIS) analysis of electrodes with 0% (D), 2.5% (E), and 5% (F) w/v glycerol (common reference formulation considered as baseline for comparisons: 3.5% w/v alginate, 5% w/v glycerol, 1 mL pDA per batch). Nyquist (left) and Bode (right) plots are shown along with the equivalent circuit models used for fitting. Experimental data are reported as black squares and fits as green solid lines.

The effect of glycerol content on the electrochemical behavior of PDA–alginate hydrogel electrodes was systematically investigated by cyclic voltammetry and electrochemical impedance spectroscopy (EIS). A representative sample is shown in **Figure** **2**A.

In the presence of 10 mm [Fe(CN)₆]^3−/4−^ (10 mm HEPES, 100 mm KCl, pH 7.2), cyclic voltammograms recorded at scan rates of 5–300 mV s^−1^ (Figure 2B) yielded anodic and cathodic peaks whose current scaled with ν^1/2^, consistent with diffusion‐controlled electron transfer. The electroactive area (A_EA_) derived from Randles–Ševčík analysis was 1.89 ± 0.05 cm^2^ without glycerol, decreased to 1.57 ± 0.06 cm^2^ at 2.5% glycerol, and increased to 1.93 ± 0.04 cm^2^ at 5% glycerol (Figure 2C). Thus, while intermediate glycerol content reduced probe accessibility, 5% glycerol restored A_EA_ to pristine levels while simultaneously improving mechanical flexibility and stability of the hydrogel matrix.

EIS spectra were analyzed using equivalent circuits shown in Figure 2D–F, incorporating solution resistance (R_S_), charge‐transfer resistances (R_CT_, R_CT2_), constant phase elements (CPEs), and a Warburg element (Z_W_) to account for semi‐infinite diffusion. The total impedance is Equation (5):

(5) $$Z\left(\omega \right)=R_{s}+\left[\frac{1}{\frac{1}{Z_{CPE1}}+\frac{1\;}{R_{CT}+Z_{W}}\;}\right]+\left[\frac{1}{\frac{1}{Z_{CPE2}}+\frac{1}{R_{CT2}}}\right]$$

where $Z_{CPE}=\frac{1}{Q\;{\left(j\omega \right)}^{n}}$ and *Z_W_* = σ (*j*ω)^−1/2^.

For the 0% glycerol electrode (Figure 2D), fitting yielded R_S_ = 1074 ± 16 Ω, R_CT_ = 13,210 ± 1334 Ω, R_CT2_ = 2639 ± 405 Ω, CPE_1_ = 45 ± 1 µF (*n* = 1.0 ± 0.1), CPE_2_ = 75 ± 4 µF (*n* = 0.8 ± 0.1), and Z_W_ = 3821 ± 385 Ω, with χ^2^ = 0.0004 (Table S2, Supporting Information). This configuration indicates two distinct charge‐transfer processes, with substantial interfacial resistance. At 2.5% glycerol (Figure 2E), R_CT_ increased markedly to 26 200 ± 2442 Ω, R_CT2_ decreased to 1487 ± 97 Ω, R_S_ dropped to 284 ± 7 Ω, while Z_W_ increased to 8921 ± 624 Ω, with χ^2^ = 0.0013 (Table S2, Supporting Information). The significant rise in R_CT_ combined with the higher Warburg impedance reflects hindered electron transfer and reduced ionic transport, consistent with the decrease in A_EA_. In contrast, the 5% glycerol electrode (Figure 2F) displayed improved transport characteristics, with R_CT_ reduced to 7730 ± 649 Ω, R_CT2_ = 955 ± 87 Ω, R_S_ = 826 ± 5 Ω, CPE_1_ = 40 ± 2 µF (*n* = 1.0 ± 0.1), CPE_2_ = 44 ± 4 µF (*n* = 0.8 ± 0.1), and Z_W_ = 4916 ± 161 Ω, with χ^2^ = 0.0001 (Table S2, Supporting Information). The simplified impedance response, reduced charge‐transfer resistance, and lower χ^2^ indicate a more homogeneous interfacial behavior and improved coupling of ionic and electronic conduction pathways.^[^ ^64^ ^]^

The Bode representations (Figure 2D–F, right panels) provided additional frequency‐dependent information on the impedance response. For the 0% w/v glycerol electrode, the phase angle approached –80° at low frequencies, consistent with capacitive control, but displayed two distinct inflection points at intermediate frequencies (10–200 Hz), reflecting the presence of two separate charge‐transfer processes (R_CT_ and R_CT2_) observed in the Nyquist fits.^[^ ^65^ ^]^ The impedance modulus increased steadily with decreasing frequency, consistent with diffusion‐limited behavior captured by the Warburg element (Z_W_ = 3821 ± 385 Ω).

At 2.5% w/v glycerol, the Bode phase plot revealed a maximum phase angle of only –60° at low frequencies, with a broader dispersion region across 1–500 Hz, indicative of increased resistive contribution and reduced capacitive dominance.^[^ ^65^ ^]^ This is consistent with the high R_CT_ (26.2 kΩ) and elevated Warburg impedance (Z_W_ = 8921 ± 624 Ω), confirming that ionic diffusion and charge transfer were strongly hindered.

In contrast, the 5% w/v glycerol electrode exhibited a phase angle again approaching –80° at low frequencies, with a sharper transition in the mid‐frequency range (10–100 Hz), consistent with a single dominant charge‐transfer process and reduced diffusion impedance (Z_W_ = 4916 ± 161 Ω). The lower R_CT_ (7.7 kΩ) and simplified phase behavior support a more homogeneous ionic–electronic transport coupling in the hydrogel matrix.^[^ ^66^ ^]^

In particular, EIS data for the self‐standing pDA–alginate electrodes, varying 0–5% w/v content of glycerol as plasticizer, were analyzed with a minimal RQ/R‐type architecture in which elements are added only when required by spectral features. The high‐frequency intercept is assigned to the bulk resistance of the hydrogel R_S_ (ionic conduction through the hydrated gel matrix, not a metal–electrolyte path). The constant‐phase elements Z_CPE_ = [Q(jω)^n^]^−1^ (Q in S·s^n^, 0≤n≤1) represent distributed double‐layer charging at the hydrogel–electrolyte boundary and within tortuous gel microdomains, which is expected for hydrated porous media and mixed ion–electron conductors.^[^ ^67^ ^]^ A second kinetic channel as a parallel (R_CT2_∥CPE) branch was introduced to explain the coexisting pathways inside the gel (e.g., less‐coupled regions and pDA‐rich domains that support redox‐hopping) in a self‐standing hydrogel electrode. A Warburg element Z_W_ = σ(jω)^−1/2^ is included in series with R_CT_ in the primary branch when the low‐frequency response exhibits the canonical diffusion signature (approach to −45° with ∣Z∣∝ω^−1/2^ and its inclusion improves AIC/χ^2^ and removes residual structure; physically, Z_W_ captures semi‐infinite diffusion of reactants/ions through the hydrogel phase (not a metal substrate).^[^ ^59^ ^]^

Overall, the data demonstrate that 5% w/v glycerol represents an optimal formulation: it preserves high electroactive area, minimizes R_CT_ compared to 0% and 2.5% w/v formulations, lowers the Warburg impedance, and improves mechanical robustness. This balance of electrochemical performance and structural stability supports its selection as the most effective glycerol concentration for pDA–alginate hydrogel electrodes.

**Figure 3:**
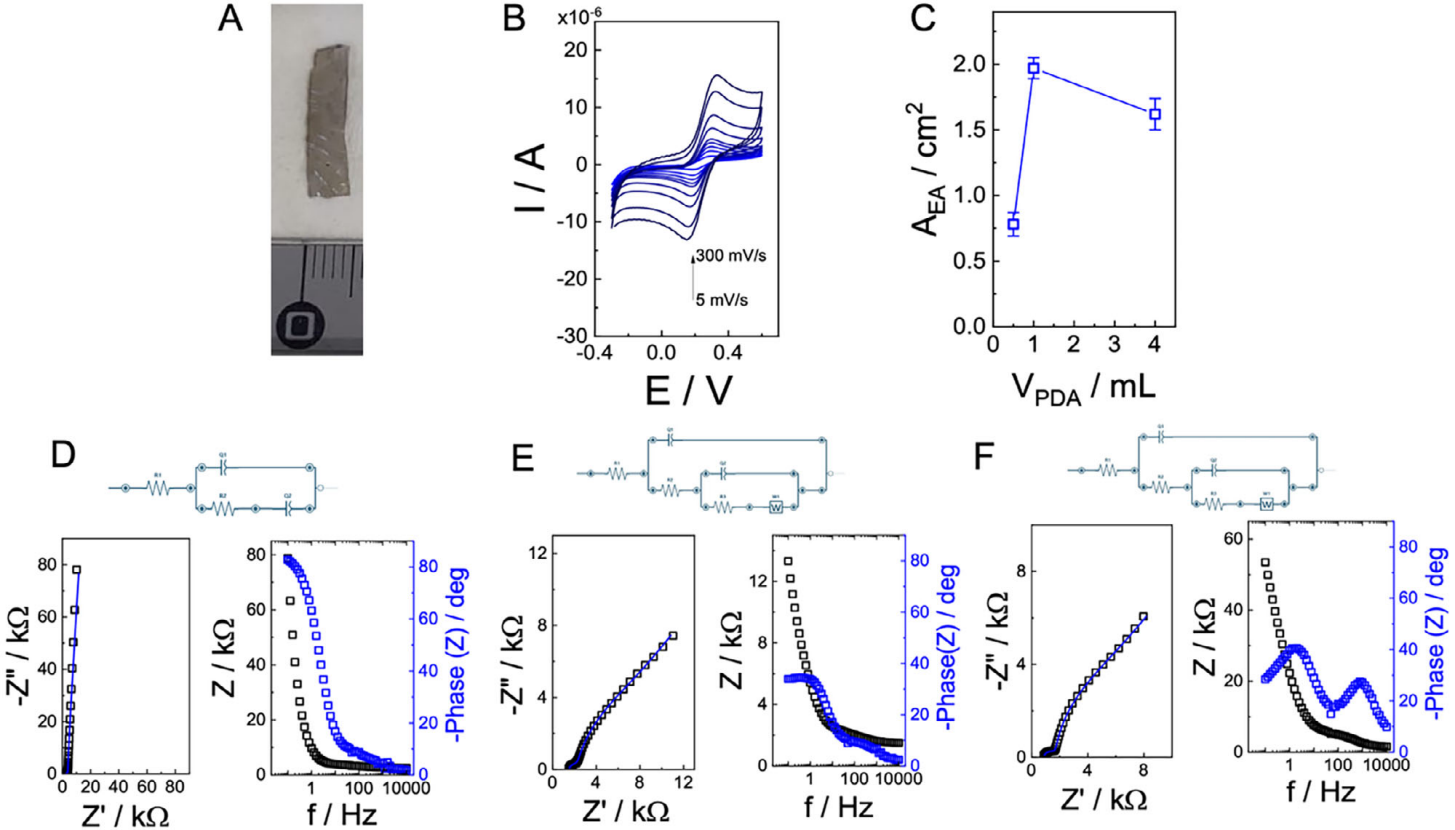
A) Photograph of a representative free‐standing hydrogel electrode (3.5% w/v alginate, 5% w/v glycerol) incorporating different pDA (1 mL). B) Cyclic voltammograms (CVs) in 10 mm HEPES buffer (pH 7.2, 100 mm KCl) with 10 mm [Fe(CN)₆]^3−^/^4−^, recorded at scan rates from 5 to 300 mV s^−1^, illustrating the effect of PDA loading on electron transfer. C) Electroactive area (A_EA_) as a function of PDA volume (0.5–4 mL), determined from Randles–Ševčík analysis of CV data. D–F) Electrochemical impedance spectroscopy (EIS) of hydrogel electrodes containing 0.5, 1, and 4 mL PDA (common reference formulation considered as baseline for comparisons: 3.5% w/v alginate, 5% w/v glycerol, 1 mL pDA per batch), respectively. Nyquist plots (left) and Bode plots (right) are presented alongside equivalent circuit models used for fitting. Experimental data are reported as black squares, with model fits shown as blue solid lines.

The effect of pDA content on the electrochemical performance of Ca^2^⁺‐crosslinked alginate hydrogel electrodes was systematically investigated. A representative free‐standing electrode is shown in **Figure** **3**A. Cyclic voltammograms recorded in the presence of 10 mm [Fe(CN)₆]^3−/4−^ (10 mm HEPES, 100 mm KCl, pH 7.2) over a scan rate range of 5–300 mV s^−1^ (Figure 3B) revealed diffusion‐controlled redox processes with anodic and cathodic peak currents proportional to ν^1/2^. The electroactive area (A_EA_), extracted from Randles–Ševčík analysis, exhibited a non‐monotonic dependence on pDA loading (Figure 3C). With 0.5 mL of pDA, the A_EA_ was significantly reduced, while incorporation of 1 mL pDA yielded the maximum value of 1.97 ± 0.08 cm^2^. Increasing pDA to 4 mL resulted in a partial decline of A_EA_, suggesting that excessive pDA reduces probe accessibility to the redox probe hindering mixed ionic‐conduction mechanisms. These results indicate that a critical pDA concentration is required to establish efficient percolation pathways while preserving ionic transport and structural homogeneity.

Electrochemical impedance spectroscopy provided further mechanistic insights. For the 0.5 mL pDA‐ electrode (Figure 3D), the Nyquist response displayed a large semicircle, consistent with sluggish interfacial kinetics. The spectrum was fitted with a model comprising solution resistance (R_S_), a constant phase element (CPE_1_), and a parallel R_CT_–CPE_2_ branch. The total impedance is expressed as Equation (6):

(6) $$Z\left(\omega \right)=R_{s}+\left[\frac{1}{\frac{1}{Z_{CPE1}}+\frac{1}{R_{CT}+Z_{CPE2}}}\right]$$

The extracted parameters were R_S_  = 2628 ± 73 Ω, R_CT_ = 1477 ± 99 Ω, CPE_1_ = 5 ± 1 µF (*n* = 0.8 ± 0.1), and CPE_2_ = 15 ± 1 µF (*n* = 1 ± 0.1), χ^2^ = 0.0006 (Table S3, Supporting Information). The Bode plot showed capacitive dominance at low frequencies but significant dispersion in the mid‐frequency region, indicative of heterogeneous interfacial charge transfer.^[^ ^65^ ^]^

At 1 and 4 mL pDA (Figure 3E,F), the Nyquist spectra required a model incorporating an additional charge‐transfer branch with Warburg diffusion. The corresponding impedance is Equation (7):

(7) $$Z\left(\omega \right)=R_{s}+\left[\frac{1}{\frac{1}{Z_{CPE1}}+\frac{1}{R_{CT}+\left(Z_{CPE2}∥\left(R_{CT2}+Z_{W}\right)\right)}}\right]$$

where Z_W_ denotes the Warburg diffusion element. The fitting parameters were R_S_ = 1455 ± 7 Ω, R_CT_ = 1193 ± 75 Ω, R_CT2_ = 7453 ± 375 Ω, CPE_1_ = 19 ± 3 µF (*n* = 0.8 ± 0.1), CPE_2_ = 29 ± 2 µF (*n* = 0.8 ± 0.1), and Z_W_ = 5811 ± 168 Ω, χ^2^ = 4.66 × 10^−5^. The reduced R_CT_ and inclusion of Z_W_ reflect the establishment of electronic percolation pathways by pDA, enabling mixed ionic–electronic conduction.^[^ ^65^ ^]^ The Bode response showed a low‐frequency phase angle approaching –80° and a mid‐frequency plateau near –45°, characteristic of combined capacitive and diffusional control.

For 4 mL pDA (Figure 3F), the spectrum was fitted with the previous equivalent circuit. The parameters were R_S_ = 783 ± 6 Ω, R_CT_ = 1182 ± 30 Ω, R_CT2_ = 5889 ± 243 Ω, CPE_1_ = 25 ± 6 µF (*n* = 0.9 ± 0.1), CPE_2_ = 43 ± 2 µF (*n* = 1 ± 0.1), and Z_W_ = 4781 ± 130 Ω, χ^2^ = 4.05 × 10^−5^. Although the solution resistance decreased due to higher pDA content, the persistence of large R_CT2_ and diffusion impedance indicates interfacial heterogeneity. The Bode plots confirmed this behavior, with broadened phase dispersion and diminished capacitive dominance relative to the 1 mL formulation.^[^ ^68^ ^]^

The equivalent circuits used in Figure 3D–F are chosen because each element represents a defined transport/charging phenomenon in a self‐standing pDA–alginate hydrogel electrode. The bulk term R_S_ is the ionic resistance of the hydrated gel phase (high‐frequency real‐axis intercept), independent of any metal–electrolyte contact. CPE_1_ with Z_CPE_ = [Q(jω)^n^]^−1^ (Q in S·s^n^, 0≤n≤1) represents distributed double‐layer charging at the gel‐electrolyte boundary and within tortuous gel microdomains; *n* < 1 quantifies the time‐constant dispersion expected for hydrated porous media.^[^ ^69^ ^]^ The primary branch (R_CT_∥CPE_2_) is the interfacial electron‐transfer route at pDA redox sites accessed by hydrated ions; R_CT_ sets the dominant semicircle diameter and the mid‐frequency phase minimum, while CPE_2_ captures non‐ideal local charging of that same route. At low pDA (0.5 mL), only this route is resolved (Eq. 6), indicating a single kinetically limiting ET pathway. At higher pDA (1 and 4 mL), a second relaxation appears because pDA reaches electronic percolation yet introduces domain heterogeneity; this is modeled by an additional kinetic channel (R_CT2_ ∥ CPE) that represents a distinct set of pDA‐rich domains/deeper regions with different ET kinetics. The Warburg element Z_W_ = σ(jω)^−1/2^ is placed in series with R_CT2_ when the low‐frequency response exhibits the ∣Z∣∝ω^−1/2^ and phase at −45° signatures, because in this configuration it physically represents semi‐infinite diffusion of reactants/ions through the gel phase feeding that second route (not mass transport in a metallic substrate).^[^ ^70^ ^]^ Thus, moving from 0.5  to 4 mL pDA, i) R_S_ tracks the ionic conductivity of pDA–alginate hydrogel electrode, ii) R_CT_ reflects changes in the primary ET exchange current as pDA connectivity increases, iii) R_CT2_ quantifies the contribution of percolated but less‐uniform domains, and iv) σ reports the diffusive impedance within the gel supplying that auxiliary route.

Overall, CV and EIS analyses demonstrate that 1 mL pDA is the optimal loading. This formulation maximizes electroactive area, reduces charge‐transfer resistance, and achieves balanced capacitive–diffusion behavior. In contrast, lower pDA‐loaded electrodes (0.5 mL) suffer from poor electronic conductivity, whereas higher pDA loadings (4 mL) introduce structural heterogeneity that impairs interfacial transport.

The intrinsic conductivity of the materials was evaluated using linear four‐point probe measurements, in which a constant current was applied through the outer probes and the potential drop was recorded across the inner probes. The measured sheet resistance (R_s_) was converted to bulk conductivity (σ = 1/(R_s_×t)), where t corresponds to the film thickness determined by profilometry (t = 100 µm), as reported in Figure S1 (Supporting Information). Pristine pDA exhibited a low conductivity of 0.35 ± 0.05 mS cm^−1^, reflecting the limited charge delocalization of the polymerized catechol network, as reported in the literature.^[^ ^71^ ^]^ Upon Ca^2^⁺ crosslinking of the pDA–alginate matrix, the conductivity increased to 4.7 ± 0.4 mS cm^−1^, evidencing the establishment of mixed ionic–electronic transport pathways mediated by catechol–Ca^2^⁺ coordination and improved interfacial charge percolation.^[^ ^72^ ^]^ The incorporation of AgNPs further enhanced conductivity to 5.3 ± 0.7 mS cm^−1^, consistent with the formation of metallic conduction bridges within the hybrid network. Subsequent GOx loading resulted in a moderate decrease to 4.4 ± 0.4 mS cm^−1^, attributable to partial obstruction of conductive channels by the insulating enzyme domains. Overall, the four‐point probe data confirm the emergence of a synergistic ionic–electronic percolation network within the self‐standing pDA–Ca^2^⁺–alginate hydrogels, enabling stable charge transport without a rigid current collector.

### 2.3 First Generation GOx/AgNPs/pDA/Ca^2+^‐Crosslinked Alginate Hydrogel Electrode: Kinetics and Analytical Parameters

**Figure 4:**
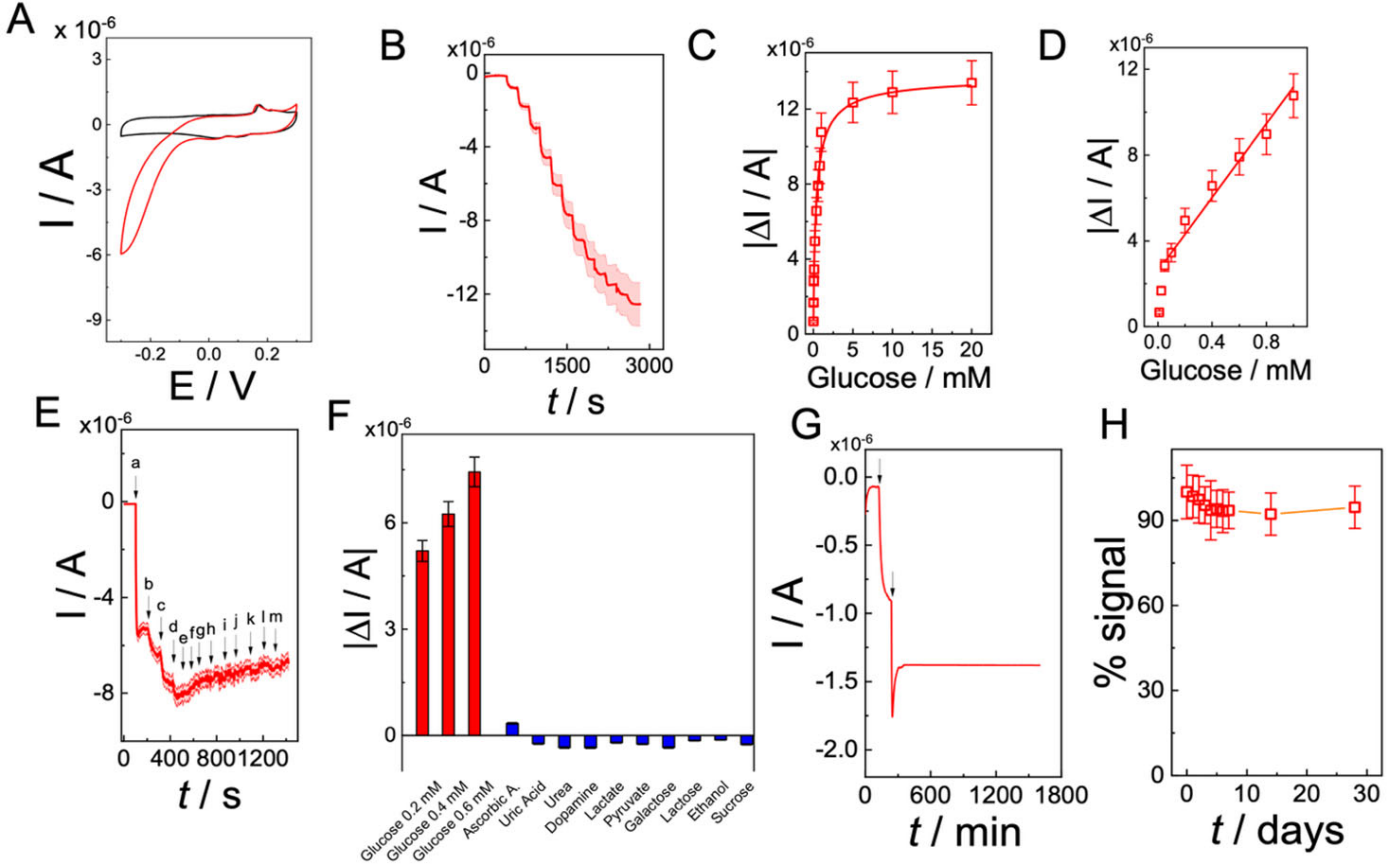
Electrochemical performance of GOx/AgNPs/pDA/Ca^2+^‐crosslinked alginate hydrogel electrode: A) Cyclic voltammograms recorded in USP‐SIF, pH 6.8, 50 mm KH_2_PO_4_ adjusted with NaOH, containing 50 mm KCl as supporting electrolyte in the absence (black) and presence (red) of 5 mm glucose; scan rate: 5 mV s^−1^. B) Amperometric response at −0.25 V in USP‐SIF, pH 6.8, 50 mm KH_2_PO_4_ adjusted with NaOH, containing 50 mm KCl as supporting electrolyte after successive glucose additions (10 µm–20 mm). C) Calibration curve of steady‐state current versus glucose concentration fitted to the Michaelis–Menten equation. D) Linear range of the calibration curve between 50 µm and 1 mm glucose. E) Amperometric response at −0.25 V in USP‐SIF, pH 6.8, 50 mm KH_2_PO_4_ adjusted with NaOH, containing 50 mm KCl as supporting electrolyte to sequential additions of glucose and potential interferents (ascorbic acid, uric acid, urea, dopamine, lactate, pyruvate, galactose, lactose, ethanol, sucrose; 0.2 mm each). F) Histogram of current responses normalized to glucose signal. G) Operational stability test during continuous amperometry at −0.25 V in USP‐SIF, pH 6.8, 50 mm KH_2_PO_4_ adjusted with NaOH, containing 50 mm KCl as supporting electrolyte with 0.1 mm glucose for 24 h. H) Storage stability test by recording the response to 0.1 mm glucose every 48 h over 28 days at 4 °C in PBS.

After optimization of the hydrogel formulation (3.5% w/v alginate, Ca^2+^ crosslinked, 5% w/v glycerol, 1 mL pDA), the material was further engineered into a first‐generation amperometric glucose biosensor by incorporation of silver nanoparticles (AgNPs), synthesized according to green chemistry protocols, as catalytic sites and food‐grade glucose oxidase (GOx) as the biorecognition element. The electrochemical behavior of the optimized GOx/AgNPs/pDA/Ca^2+^‐crosslinked alginate hydrogel electrode was first evaluated by cyclic voltammetry at 5 mV s^−1^ in USP Simulated Intestinal Fluid (SIF, pH 6.8). As shown in **Figure** **4**A, in the absence of glucose (black curve), the electrode exhibited a pair of well‐defined anodic and cathodic peaks with a formal potential of E°′ = 0.168 V vs Ag/AgCl(sat), assigned to the reversible redox activity of AgNPs embedded within the hydrogel matrix. The sharp and symmetric features of these peaks confirm the electrochemical accessibility of the nanoparticles, their stability within the composite scaffold, and the absence of significant passivation processes typically observed on bare silver surfaces.^[^ ^73^ ^]^ Upon addition of glucose (5 mm), a pronounced catalytic cathodic wave developed (red curve), attributable to the enzymatic oxidation of glucose by GOx with in situ generation of H_2_O_2_, which was efficiently reduced at AgNPs catalytic sites. The catalytic current displayed an onset potential of +0.05 V vs Ag/AgCl(sat) and reached a maximum of −5.9 ± 0.2 µA at −0.30 V, highlighting the role of AgNPs in lowering the overpotential for H_2_O_2_ reduction and thereby enhancing the sensitivity of the biosensor.

The modified GOx/AgNPs/pDA/Ca^2+^‐crosslinked alginate hydrogel electrode was tested in amperometry at E = −0.25 V vs Ag/AgCl(sat) by increasing glucose concentration in the range 0–20 mm, as reported in Figure 4B.

The calibration curve for GOx/AgNPs/pDA/Ca^2+^‐crosslinked alginate hydrogel electrodes in USP SIF (pH 6.8) is reported in Figure 4C, covering the concentration range 1 × 10^−5^ to 2 × 10^−2^ M. The response was linear from 50 µm to 1 mm (Figure 4D), with a detection limit of 10.4 µm and a sensitivity of 8.6 ± 0.6 µA mm ^−1^ (normalized by area 34.4 µA mm ^−1^ cm^−2^, RSD 6.9%, *n* = 20), and a correlation coefficient of 0.989. The steady‐state catalytic currents were fitted according to the Michaelis–Menten model (Equation 8):

(8) $$I\left(C\right)=\frac{I_{max}\left[C\right]}{K_{M}^{app}+\left[C\right]}$$

where I(C) is the current at substrate concentration C, I_max_ is the maximum catalytic current, and K_M_^app^ is the apparent Michaelis–Menten constant.^[^ ^74^ ^]^ Nonlinear regression yielded I_max_ = 13.8 ± 0.9 µA and K_M_^app^ = 0.35 ± 0.08 mm, suggesting that enzymatic activity was retained in the hydrated matrix, with deviations from solution values attributable to diffusion and partitioning constraints within the hydrogel scaffold.

The selectivity of GOx/AgNPs/pDA/Ca^2+^‐crosslinked alginate hydrogel electrode was further investigated by amperometry in USP SIF supplemented with common intestinal interferents (ascorbic acid, uric acid, dopamine, fructose, galactose, sucrose, lactate). Figure 4E shows that glucose additions at 0.2, 0.4, and 0.6 mm elicited clear stepwise increases in cathodic current, while interferents generated negligible responses. The comparative analysis reported in Figure 4F indicates that interferent signals did not exceed 0.8% of the glucose signal, except for ascorbic acid, which produced 4.3% of the glucose response. This effect can be attributed to its relatively high diffusion coefficient (D = 5.9 × 10^−6^ cm^2^ s^−1^) and facile electro‐oxidation at the working potential, consistent with observations in other electrochemical biosensing systems.^[^ ^75^ ^]^

Operational stability was assessed under continuous turnover at −0.25 V in USP SIF containing 0.1 mm glucose (Figure 4G). The electrode delivered a steady current of −1.4 ± 0.2 µA over 1370 min, with a drift of only 0.2% h^−1^, confirming stable charge transport through the pDA–AgNPs domains. Storage stability (Figure 4H) was evaluated over 30 days at 4 °C, showing retention of ≥90% of the initial signal after 20 days and 94.5% after 30 days, highlighting the protective effect of the alginate–pDA matrix on both enzyme and nanoparticles.

Overall, the analytical figures of merit (LOD, linear range, sensitivity, I_max_, and K_M_^app^) are summarized in Table S4 (Supporting Information). The edible GOx/AgNPs/pDA/Ca^2+^‐crosslinked alginate hydrogel electrode combines efficient electrocatalysis of H_2_O_2_ with long‐term stability and selectivity in USP SIF, achieving analytical performance similar to conventional solid‐state biosensors while uniquely enabling safe gastrointestinal operation.^[^ ^76^, ^77^ ^]^

Moreover, the mussel‐inspired chemistry introduced by polydopamine (pDA) plays a pivotal role in maintaining both the electrochemical performance and structural integrity of the hydrogel electrode. In this context, “mussel‐inspired” denotes the catechol‐based coordination and redox chemistry intrinsic to pDA, which strengthens the Ca^2^⁺–alginate network through catechol–Ca^2^⁺ complexation and covalent Schiff‐base/Michael addition reactions with enzyme amines.^[^ ^30^ ^]^ These interactions markedly reduce enzyme leakage (increasing of 83% for payload retention after 6 h) and establish stable mixed ionic–electronic conduction pathways, ensuring reproducible signal transduction over time.^[^ ^78^ ^]^ As confirmed by the enzyme‐leakage analysis (Figure S2, Supporting Information), Ca^2^⁺–alginate hydrogels progressively released the enzyme up to 95 ± 4% of its initial payload, i.e., GOx labeled with, whereas the pDA–Ca^2^⁺–alginate formulation exhibited negligible loss up to 12 ± 1%, correlating with its electrochemical stability and retained sensing response.

The pDA–Ca^2^⁺–alginate–AgNPs–GOx electrode exhibits a LoD of 10.4 µm, linear range of 50 µm–1 mm, and sensitivity of 34.4 µA mm ^−1^ cm^−2^ at −0.25 V vs Ag/AgCl, achieving efficient glucose detection under mild potentials (Table S5, Supporting Information). Compared to PtCo nanozyme/GOx,^[^ ^79^ ^]^ PtNPs–chitosan/GOx,^[^ ^80^ ^]^ and silk‐fibroin/GOx^[^ ^81^ ^]^ systems, which operate at higher anodic/cathodic potentials (−0.30 to +0.80 V) and exhibit higher detection limits, the present edible electrode delivers superior low‐potential response through mixed ionic–electronic conduction within the pDA–Ca^2^⁺–alginate–AgNPs matrix, eliminating the need for metallic current collectors.

### 2.4 Rheological, SAXS, and Mechanical Characterization of GOx/AgNPs/pDA/Ca^2+^‐Crosslinked Alginate Hydrogel Electrode

To gain insight into the structural organization and mechanical behavior of the pDA–alginate hydrogel systems, we performed a multiscale physico‐chemical characterization combining oscillatory shear rheology, small‐angle X‐ray scattering (SAXS), and dynamic mechanical analysis (DMA). Rheology was employed to probe the viscoelastic properties of the precursor solutions used for film preparation, SAXS provided information on nanoscale structuring and particle–matrix interactions, while DMA enabled evaluation of the bulk mechanical response of the crosslinked hydrogels under simulated physiological conditions.

The shear rheology provides information on the initial solutions adopted for the film preparation. From the frequency sweep measurements, it is clearly observed that for all the analyzed samples, the viscous modulus G″ is predominant over the elastic modulus G′ across the entire tested frequency range (Figure S3A, Supporting Information). This behavior indicates that the analyzed systems have a viscoelastic component dominated by viscosity, and therefore, they are considered primarily viscous. The samples containing silver nanoparticles (AgNPs), with or without glucose oxidase (GOx), show significantly higher absolute values of G’ and G″ compared to the other systems, and their values are very similar to each other, with a measurable G’ even at relatively high frequencies. This suggests a greater degree of internal structuring in these systems, likely due to the interaction between the nanoparticles and the matrix. This observation is consistent with the results obtained from the flow curves (Figure S3B, Supporting Information), from which, by fitting with the Carreau model, the zero‐shear viscosity values can be extracted. All systems behave as pseudoplastic fluids, but those containing AgNPs (with or without GOx) display a zero‐shear viscosity an order of magnitude higher than systems without nanoparticles. The introduction of AgNPs and, even more, the combination of AgNPs with glucose oxidase (GOx), leads to an increase in the viscoelastic moduli (both G' and G″), suggesting greater internal organization and interactions between the particles.

**Figure 5:**
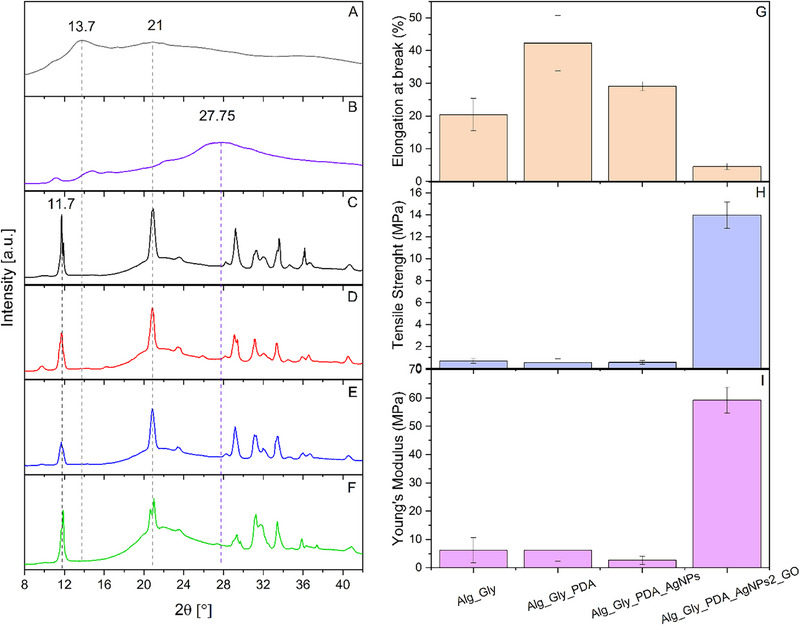
WAXS patterns for alginate powder (A), pDA powder (B), alginate_glycerol (C), alginate_glycerol_pDA (D), alginate_glycerol_pDA in the presence of AgNPs (E), and alginate_glycerol_pDA in the presence of AgNPs with GOx (F). Dynamic mechanical analysis of alginate‐based electrodes: elongation at break (G), tensile strength (H), and Young's modulus (I).

Wide‐angle X‐ray scattering (WAXS) analysis was carried out to assess the structural organization of the alginate‐based hydrogel electrodes and the influence of pDA, AgNPs, and GOx on crystallinity (**Figure** **5**A–F). Sodium alginate is characterized by broad peaks at 2θ = 13.7° and 21.8°, which correspond to the lateral packing of alginate chains and intrachain spacing, respectively.^[^ ^82^, ^83^ ^]^ In the pristine alginate/glycerol hydrogel electrodes (Figure 5A), two broad peaks centered at 13.7° and 21° were observed, confirming the predominantly amorphous nature of the system. pDA exhibited a broad reflection peak at 27.75° (Figure 5B), consistent with its amorphous carbonaceous structure, which remained largely unchanged after incorporation into the hydrogel network.^[^ ^84^ ^]^ Upon ionic crosslinking with Ca^2^⁺, the alginate hydrogel electrodes showed the emergence of sharper reflections, most notably a peak at 11.7° (d‐spacing ≈ 7.6 Å), confirming the formation of semicrystalline domains (Figure 5C). The feature at 21° appeared as a sharp diffraction peak superimposed on a broad halo, suggesting coexistence of amorphous and crystalline regions. Introduction of pDA (Figure 5D) and AgNPs (Figure 5E) did not significantly perturb this semicrystalline organization, whereas incorporation of GOx (Figure 5F) induced a marked increase in the amorphous contribution and a splitting of the 21° peak. The disappearance of the GOx (001) reflection, typically reported at 2θ ≈ 14.5°, indicates disruption of its layered structure and possible intercalation or exfoliation within the alginate–pDA matrix.^[^ ^85^ ^]^

Mechanical characterization through tensile‐type experiments using Dynamic Mechanical Analysis (DMA) further corroborated the structural findings. The elongation at break (Figure 2G) ranged from 20% to 40% for formulations containing alginate, pDA, and AgNPs, but dropped sharply to ≈5% upon incorporation of GOx, indicating a brittle fracture behavior. Conversely, tensile strength (Figure 5H) and Young's modulus (Figure 5I) were significantly enhanced in the GOx‐containing electrodes, reaching values of ≈14 and ≈65 MPa, respectively, compared to <1 MPa for the other systems. These results demonstrate that while pDA and AgNPs preserve the semicrystalline structure and elasticity of the films, the inclusion of GOx disrupts crystallinity, increases amorphous content, and reinforces stiffness and tensile strength, likely due to strong interfacial interactions with the polymer matrix.

### 2.5 Morphological (SEM, TEM, and AFM) and FTIR Characterization of GOx/AgNPs/pDA/Ca^2+^‐Crosslinked Alginate Hydrogel Electrode

**Figure 6:**
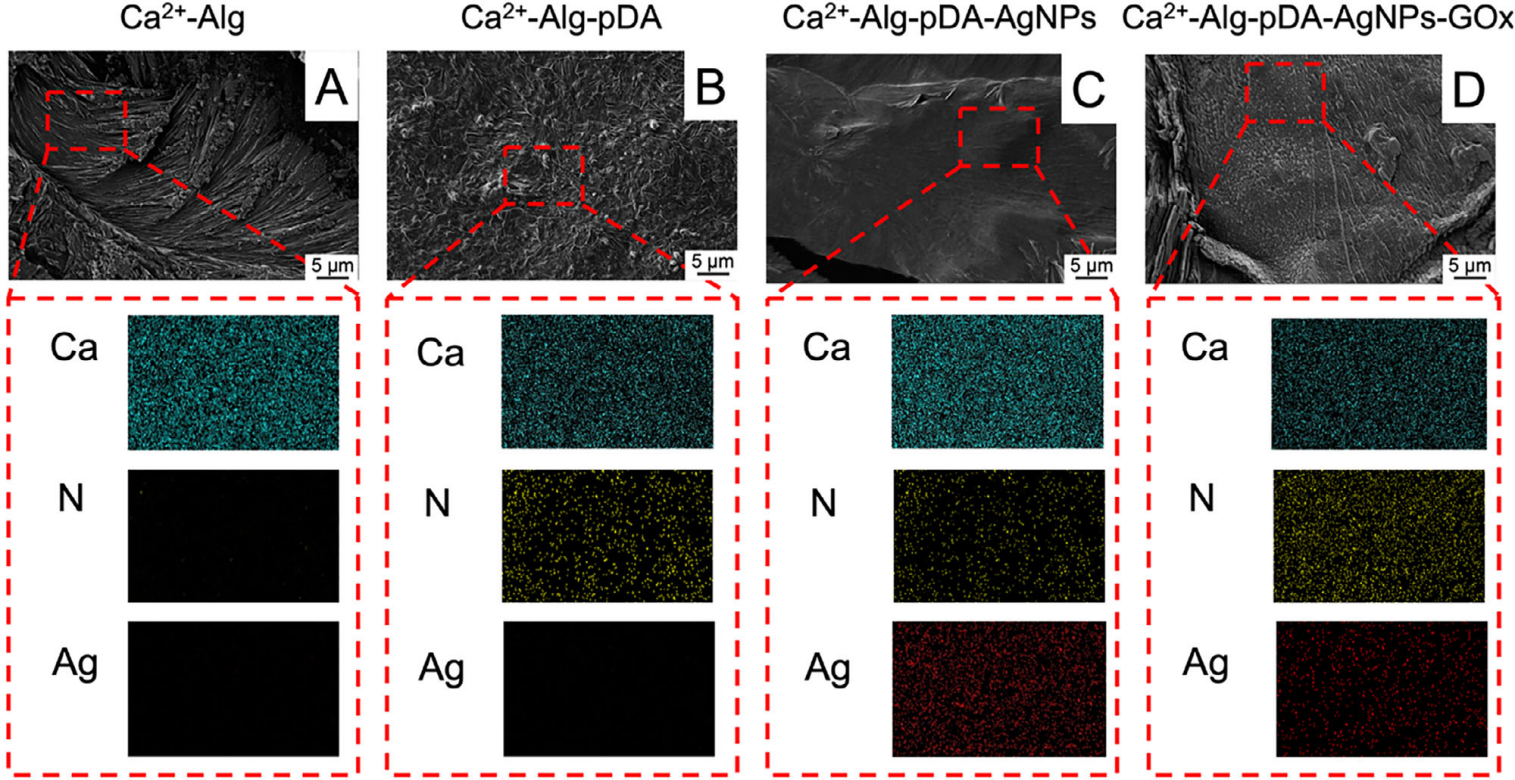
SEM micrographs (top panels, scale bar = 5 µm) and EDX elemental mapping (bottom panels) for: (A) Ca^2^⁺–Alg, (B) Ca^2^⁺–Alg–pDA, (C) Ca^2^⁺–Alg–pDA–AgNPs, (D) Ca^2^⁺–Alg–pDA–AgNPs–GOx. Elemental maps display Ca (cyan), N (yellow), and Ag (red).

The morphological and compositional characteristics of the hydrogel films were investigated by scanning electron microscopy (SEM) coupled with energy‐dispersive X‐ray spectroscopy (EDX) elemental mapping (**Figure** **6**A–D).^[^ ^86^ ^]^ The Ca^2^⁺–crosslinked alginate matrix (Figure 6A) exhibited a fibrous morphology with a lamellar‐like texture typical of ionically gelled polysaccharides. Elemental mapping confirmed the homogeneous distribution of calcium throughout the structure, consistent with efficient crosslinking, while no nitrogen or silver signals were detected. Incorporation of polydopamine (Figure 6B) led to a denser and smoother surface morphology, indicative of enhanced interfacial adhesion between polymer chains mediated by catechol–amine chemistry. The presence of uniformly distributed nitrogen confirmed the successful incorporation of pDA within the matrix. Upon embedding AgNPs (Figure 6C), SEM micrographs revealed the appearance of finer and more compact domains, while EDX mapping showed a homogeneous distribution of silver throughout the hydrogel network, in addition to calcium and nitrogen signals. Finally, the co‐immobilization of GOx together with AgNPs (Figure 6D) did not substantially alter the bulk morphology, but the elemental maps displayed simultaneous and uniform distribution of Ca, N, and Ag, confirming effective incorporation of all functional components. The absence of aggregation or clustering of AgNPs in the mapping highlights their stable dispersion within the alginate–PDA matrix, a critical feature for ensuring reproducible electrochemical activity and consistent catalytic performance in biosensing applications.

Transmission electron microscopy (TEM) was employed to examine the morphology, size distribution, and crystalline features of the AgNPs (Figure S4, Supporting Information). The micrograph reveals a heterogeneous nanoparticle population dominated by spherical particles, accompanied by triangular and polygonal nanostructures, indicative of the coexistence of multiple crystallographic growth directions. The particle dimensions spanned ≈14–57 nm, with an average diameter of 34 ± 4 nm, confirming nanoscale dispersion suitable for electrocatalytic applications. The observation of triangular nanoplates and faceted morphologies highlights preferential growth along specific crystallographic planes, particularly high‐energy {111} facets, which are well known to enhance catalytic activity toward hydrogen peroxide reduction.^[^ ^87^ ^]^

The AFM images reported in Figure S5 (Supporting Information) highlight marked differences in the surface morphology between Ca^2+^‐crosslinked alginate electrodes (Figure S5A, Supporting Information) and those additionally containing pDA (Figure S5B, Supporting Information). The sample composed of pure alginate exhibited a uniform and finely structured surface, with roughness in the nanometer range (−1.8 to +2.0 nm). The texture appeared compact and regular, consistent with the SEM observation (Figure 6A), which revealed a relatively smooth and striated surface resulting from the drying process and ionic crosslinking. Similarly, the inclusion of polydopamine within the matrix (Figure S5B, Supporting Information) induced an increasing roughness and the development of a complex 3D architecture, with topographic variations reaching ±260 nm.^[^ ^86^ ^]^

The FTIR spectra reported in Figure S6 (Supporting Information) illustrate the progressive chemical evolution of the alginate‐based hydrogel matrix (3.5% w/v alginate, 5% w/v glycerol) following the sequential incorporation of polydopamine (pDA), silver nanoparticles (AgNPs), and glucose oxidase (GOx). The baseline system (green spectrum), composed of alginate and glycerol, displayed the characteristic vibrational features of alginate, including the asymmetric and symmetric stretching of carboxylate groups (─COO^−^) at ≈1595 and 1410 cm^−1^, respectively, together with a broad absorption band between 3200 and 3400 cm^−1^ associated with O─H stretching.^[^ ^88^ ^]^ Upon addition of 1 mL of pDA (blue spectrum), spectral changes were observed, most notably an increased intensity in the 1510–1600 cm^−1^ region, attributable to C═C vibrations of the aromatic ring and N─H bending modes of PDA,^[^ ^88^ ^]^ suggesting interactions between amine groups of pDA and the carboxylate moieties of alginate. Incorporation of AgNPs (red spectrum) did not drastically modify the main band profile but induced subtle shifts and variations in band intensity, which may be associated with coordination phenomena between AgNPs and functional groups of the hydrogel network. Finally, the introduction of GOx (black spectrum) produced a marked spectral enrichment, with the appearance of additional bands in the 1000–1200 cm^−1^ region, characteristic of C─O─C and C─N vibrations in protein structures, and a broadening of the O–H/N–H stretching band ≈3300 cm^−1^, consistent with the integration of the enzyme into the polymeric scaffold.

Overall, the FTIR analysis confirms the successful and progressive integration of pDA, AgNPs, and GOx into the alginate hydrogel, highlighting chemical and physical interactions among the functional components within the crosslinked polymeric network.

### 2.6 MTT Assay to Assess the Cytotoxicity of the Hydrogels Toward Caco‐2 Cells

The MTT assay was performed to assess the cytotoxicity of the hydrogels toward Caco‐2 cells.

After 24 h of exposure, no significant decrease in cell viability was observed in any of the tested groups compared to the untreated control, as reported in Figure S7 (Supporting Information). Cell viability confirmed the absence of acute cytotoxic effects.

The control group showed a normalized viability of 100%, which was used as a reference for comparison. The Ca^2^⁺–Alg group exhibited a slight reduction in cell viability, reaching ≈70%, corresponding to a 30% decrease compared to the control. This mild reduction could be attributed to possible ionic interactions or osmotic effects associated with calcium alginate matrices.

The introduction of polydopamine led to a partial recovery of cell viability, reaching ≈85%, which represents a 15% decrease compared to the control but a ≈21% increase compared to Ca^2^⁺–Alg group. This suggests that the polydopamine may mitigate some of the cytotoxic effects by improving the surface compatibility and promoting better cell adhesion.

Finally, the sample containing silver nanoparticles exhibited a cell viability close to 100%, showing almost no reduction compared to the control group. This result highlights that the incorporation of AgNPs, when properly stabilized within the pDA‐modified hydrogel matrix, does not induce cytotoxic effects at the tested concentrations. Instead, the combination of pDA and AgNPs appears to preserve cellular integrity, possibly due to a synergistic effect between surface modification and nanoparticle stabilization that prevents uncontrolled silver ion release.

These results indicate good initial biocompatibility of the developed hydrogels, supporting their potential for further biological evaluations.

## 3 Conclusion

This study reports for the first time the development of a novel self‐standing, edible soft electrodes fabricated from Ca^2^⁺‐crosslinked alginate hydrogels (3.5% w/v) plasticized with glycerol (5% w/v) and reinforced with 1 mL of polydopamine (pDA). The optimized formulation exhibited an electroactive surface area of 1.99 ± 0.07 cm^2^ and a double‐layer capacitance of 10.1 ± 0.3 µF, as determined from cyclic voltammetry and non‐faradaic charging curves. Electrochemical impedance spectroscopy revealed a solution resistance (R_S_) of 826 ± 5 Ω and a reduced charge‐transfer resistance (R_CT_) of 7.7 ± 0.6 kΩ compared to lower alginate contents, while maintaining a Warburg element of ≈4.9 kΩ, confirming efficient ionic diffusion within the hydrogel network.

Rheological analysis showed that AgNPs‐ and GOx‐containing samples displayed a ten‐fold higher zero‐shear viscosity compared to pristine hydrogels, consistent with greater internal structuring, while DMA measurements reported a tensile strength of ≈14 MPa and a Young's modulus of ≈65 MPa in GOx‐containing electrodes, versus <1 MPa in unmodified alginate. WAXS and the corresponding analysis confirmed crystalline ordering consistent with a homogeneous dispersion of nanoparticles. AFM revealed a surface roughness increase from ≈±2 nm in pristine alginate to ±260 nm upon pDA incorporation. SEM and TEM further validated uniform AgNPs dispersion with particle dimensions in the 20–50 nm range. FTIR spectroscopy confirmed the progressive integration of PDA (1510–1600 cm^−1^ aromatic and N─H vibrations), AgNPs coordination with carboxylates, and protein incorporation (1000–1200 cm^−1^ C─O─C/C─N modes).

Configured as a first‐generation glucose biosensor operating in USP simulated intestinal fluid (pH 6.8), the platform exhibited a linear range of 50 µm–1 mm, a limit of detection (LOD) of 10.4 µm, and a sensitivity of 8.6 ± 0.6 µA mm ^−1^ cm^−2^ (R^2^ = 0.989, *n* = 20). The catalytic current followed Michaelis–Menten kinetics with I_max_ = 13.8 ± 0.6 µA and an apparent K_M_^app^ = 0.35 ± 0.08, comparable to solution‐phase values. The biosensor retained ≥95% of its initial activity after 20 h of continuous turnover and 90% after 30 days of storage at 4 °C, demonstrating both operational and long‐term stability. Interferent analysis revealed negligible current contributions (<0.8% of glucose signal) for fructose, galactose, dopamine, and uric acid, while ascorbic acid generated ≈4% of the glucose response, consistent with its high diffusivity (D = 5.9 × 10^−6^ cm^2^ s^−1^) and electroactivity at the applied potential.

The maintained high cell viability also suggests that the material composition and preparation process do not release harmful by‐products within the first 24 h of contact, a crucial prerequisite for in vivo applications and for sustained therapeutic delivery.

Overall, these findings demonstrate that GOx/AgNPs/pDA/Ca^2+^‐crosslinked alginate hydrogel electrode combine mechanical robustness, nanoscale organization, and electrochemical activity into a biodegradable, edible format, achieving analytical performance comparable to conventional solid‐state biosensors while uniquely enabling safe, transient, and selective glucose monitoring within the gastrointestinal environment. This work establishes a quantitative foundation for the development of next‐generation ingestible bioelectronic devices for metabolic monitoring and personalized diabetes management.

## 4 Experimental Section

### Chemicals and Materials

Sodium alginate (from brown algae, medium viscosity), calcium chloride dihydrate (CaCl_2_·2H_2_O, ≥99%), glycerol (≥99.5%), dopamine hydrochloride (≥98%), Trizma® hydrochloride (Tris‐HCl, ≥99%), HEPES (≥99.5%), potassium ferricyanide (K_3_[Fe(CN)₆], 99%), potassium ferrocyanide trihydrate (K_4_[Fe(CN)₆]·3H_2_O, 99%), potassium chloride (KCl, ≥85%), sodium sulfate (Na_2_SO_4_, ≥99%), silver nitrate (AgNO_3_, ≥99%), quercetin (3,3′,4′,5,7‐pentahydroxyflavone, ≥98%), hydrogen peroxide (H_2_O_2_, 30% w/v solution), D‐(+)‐glucose (food‐grade, ≥99.5%), sodium hydroxide (NaOH, ≥98%), hydrochloric acid (HCl, 37%), monobasic potassium phosphate (KH_2_PO_4_, ≥99%), and all other salts were purchased from Merck (formerly Sigma–Aldrich, Darmstadt, Germany) and used as received without further purification. Food‐grade glucose oxidase (GOx, ≥100 U mg^−1^, from *Aspergillus niger*) was obtained from Amano Enzyme (Nagoya, Japan). Deionized water (18.2 MΩ·cm, Milli‐Q system, Millipore) was used throughout all preparations and electrochemical measurements.

### Electrode Preparation

Hydrogel electrodes were fabricated by dissolving sodium alginate (3.5% w/v) in 100 mm Na_2_SO_4_ solution at 45 °C under vigorous stirring for 24 h. Glycerol (5% w/v) was added as a plasticizer.^[^ ^24^ ^]^ Polydopamine (pDA) was synthesized by oxidative self‐polymerization of dopamine hydrochloride (2 mg mL^−1^) in 10 mm Tris buffer (pH 8.6) under continuous stirring (600 rpm) for 6 h at room temperature, yielding a dark suspension.^[^ ^89^ ^]^ A 1 mL aliquot of pDA was incorporated into the alginate–glycerol solution, followed by addition of AgNPs (3 mm, prepared as below) and glucose oxidase (GOx, 0.2 g).

Aliquots (2 mL) of the composite solution were cast onto 6 cm glass Petri dishes, crosslinked by immersion in CaCl_2_ (30 mm) for 60 min, and dried at 35 °C for 30 min. The resulting free‐standing films (1.5 × 0.5 cm, active area 0.25 cm^2^) were used as electrodes without rigid supports.

### Electrode Preparation—Synthesis of Silver Nanoparticles (AgNPs)

AgNPs were prepared by reduction of AgNO_3_ (10 mm) in HEPES buffer (100 mm, pH 7.0) using quercetin (1 mm in NaOH 1 M) as a green reducing agent. Briefly, 6.5 mL HEPES, 3 mL AgNO_3_, and 0.5 mL quercetin solution were sequentially mixed under stirring. The reaction mixture was protected from light and stirred for 30 min, yielding a colloidal AgNP suspension (278 µm). The colloid was directly incorporated into hydrogel formulations.^[^ ^87^ ^]^

### Electrode Preparation—Preparation of USP Simulated Intestinal Fluid (USP SIF)

USP SIF without enzymes was prepared according to the pharmacopeia protocol^[^ ^90^ ^]^ by dissolving 6.8 g monobasic potassium phosphate in 250 mL water, adjusting pH to 6.8 with 0.2 N NaOH, and diluting to 1000 mL with water. The final solution (pH 6.8 ± 0.05) was used as electrolyte for glucose biosensing.

### Electrochemical Characterization

Electrochemical experiments were performed using a PalmSens MultiPalmSens4 potentiostat (PalmSens, The Netherlands) in a standard three‐electrode configuration, employing the hydrogel‐based electrode as the working electrode, a Pt wire as counter electrode, and an Ag/AgCl electrode (saturated KCl, +0.198 V vs NHE) as reference.

### Electrochemical Characterization—Cyclic Voltammetry (CV)

Measurements were conducted in HEPES buffer (10 mm, pH 7.2) containing 100 mm KCl, in the absence or presence of 10 mm [Fe(CN)₆]^3−^/^4−^ as a redox probe. Voltammograms were acquired in the potential range from –0.3 to +0.6 V, with scan rates varied between 5 and 300 mV s^−1^. The electroactive area (A_EA_) was calculated by applying the Randles–Ševčík equation to the peak currents.

### Electrochemical Characterization—Electrochemical Impedance Spectroscopy (EIS)

EIS measurements were carried out at open‐circuit potential, over a frequency range from 10 kHz to 0.1 Hz, with a sinusoidal perturbation amplitude of 10 mV. The Nyquist and Bode plots were analyzed using Zview v.4 software, and data were fitted with equivalent circuit models containing solution resistance (R_S_), charge‐transfer resistance (R_CT_), constant phase elements (CPEs), and, when appropriate, a Warburg diffusion element (Z_W_).^[^ ^65^ ^]^

### Electrochemical Characterization—Amperometry

Amperometric detection was performed in USP simulated intestinal fluid (USP SIF, pH 6.8) under constant potential at –0.25 V. Glucose additions were carried out stepwise in the concentration range 50 µm–20 mm. Calibration plots were constructed from steady‐state current responses and fitted to the Michaelis–Menten equation:

(9) $$I\left(C\right)=\frac{I_{max}\left[C\right]}{K_{M}^{app}+\left[C\right]}$$

where I(C) is the current at substrate concentration C, I_max_ is the maximum catalytic current, and K_M_^app^ is the apparent Michaelis–Menten constant.

### Conductivity Measurements

Linear four‐point probe measurements were performed to determine the bulk conductivity of self‐standing hydrogel films using a probe spacing (s) of 1 mm. A constant DC current (I) was applied through the outer probes, while the potential drop (ΔV) between the inner probes was recorded using a high‐impedance voltmeter to eliminate contact resistance effects. The sheet resistance (R_s_) was calculated as Rs = (π/ln(2)) (ΔV/I) for an infinite sheet geometry, and the bulk conductivity (σ) was obtained from σ = 1/(Rs×t), where t is the film thickness measured by profilometry. All measurements were performed under ambient conditions using at least three independent samples per formulation.

### Glucose Oxidase Leakage Measurements

Fluorescence was recorded on a Thermo Scientific Varioskan LUX multimode plate reader (monochromator‐based), top‐read, black 96‐well plates (100 µL per well, lid on). Parameters: λ_ex = 485 ± 5 nm, λ_em = 520 ± 5 nm (both 5 nm slits). Temperature control: 37 °C (release kinetics); orbital shaking 300 rpm for 5 s before each read. For fluorescence‐based release experiments, GOx was covalently labeled with fluorescein isothiocyanate (FITC) through amine coupling. FITC was dissolved in 50 mM carbonate–bicarbonate buffer (CBB, pH 9.0) at a final concentration of 1 mg mL^−1^, and 200 µL of this solution was added dropwise to 1 mL of protein or enzyme solution (3 mg mL^−1^ in CBB, pH 9.0) under continuous stirring. The reaction proceeded for 12 h at 4 °C in the dark to prevent photobleaching. The labeled products were purified by size‐exclusion chromatography (Sephadex G‐50) using 25 mm HEPES buffer (pH 7.4) as eluent. The first fluorescent fractions were collected, freeze‐dried, and redissolved in 25 mm HEPES buffer (pH 7.4) to yield FITC–protein (20 mg mL^−1^) or FITC–enzyme (5 mg mL^−1^) stock solutions for subsequent loading into the hydrogel matrices.

### Rheological, X‐Ray Diffraction (XRD), and Dynamic Mechanical Analysis (DMA)

Rheological measurements were carried out using an Anton Paar MCR 302 Evolution (Anton Paar, Graz, Austria) equipped with a Couette geometry (double concentric cylinder). The temperature was kept constant at 25 °C by means of a Peltier‐controlled system, connected to a water circulator as a thermal reference. Oscillatory frequency sweep tests were performed within the linear viscoelastic region to determine the storage modulus G′ (elastic modulus) and the loss modulus G″ (viscous modulus). Rotational measurements were interpreted using the Carreau–Yasuda model,^[^ ^91^ ^]^ which was adopted as follows:

(10) $$\eta =\frac{\eta_{0}-\eta_{\infty }}{{\left(1+{\left(\lambda \dot{\gamma }\right)}^{a}\right)}^{\left(1-n\right)/a}}+\eta_{\infty }$$

where *a* is the parameter that determines the curvature of the transition region between the Newtonian regime at low shear rates (when *a* = 2, the model reduces to the Carreau model) and the power‐law regime; *n* is the flow index; *λ* had the dimensions of time and represents a characteristic time of the system.

### Wide‐Angle X‐Ray Scattering (WAXS)

The crystalline structure of both thin films and powders using the SAXSpoint 2.0 instrument (Anton Paar GmbH, Graz, Austria), a Small‐ and Wide‐Angle X‐ray Scattering (SAXS/WAXS) system. The WAXS pattern of semicrystalline materials might also be referred as X‐diffraction pattern (XRD). The SAXSpoint 2.0 was equipped with a Primux 100 microfocus X‐ray source (Cu Kα radiation, λ = 0.15418 nm, 50 W), and the scattering intensity 𝐼(𝑞) was recorded using a Dectris EIGER R 1M detector (Dectris Ltd, Baden, Switzerland) with a pixel size of 75 × 75 µm^2^. Data were acquired with the EIGER detector mounted in a tilted configuration for wide‐angle measurements (e.g., XRD), covering a 2θ range from 8° to 42°. The sample temperature was maintained at 25 °C using a solid sample holder.

The alginate film samples were analyzed through tensile DMA measurements using a single motor stress‐controlled Anton Paar MCR 702 Evolution rheometer (Anton Paar, Graz, Austria) equipped with solid rectangular fixture (SRF).^[^ ^92^ ^]^ The specimen dimensions were, on average, 40 ± 2 mm length, 10 ± 1 mm width, and 60 ± 4 µm in thickness for all samples.

### Scanning Electron Microscopy (SEM), Transmission Electron Microscopy (TEM), Atomic Force Microscopy (AFM), and Attenuated Total Reflection–Fourier Transform Infrared Spectroscopy (ATR‐FTIR)

The morphological characteristics of all samples were analyzed using a field emission scanning electron microscope (FE‐SEM), model Leo 1530 (ZEISS, Jena, Germany).^[^ ^93^ ^]^ The microscope was operated at a low voltage of 3.00 kV to minimize charging effects and prevent thermal damage to the dielectric polymer. For elemental analysis and mapping, Energy Dispersive X‐ray Spectroscopy (EDS) was performed with an X‐MAX detector (AZTEC, Oxford, UK). The SEM operating voltage was increased to 5 kV during EDX measurements to enhance the detection of elemental peaks in the spectra. EDX was used to identify and map the spatial distribution of nitrogen (N), silver (Ag), and Calcium (Ca) in the samples.

AgNPs were deposited by drop‐casting colloidal suspensions onto carbon‐coated copper grids and dried under ambient conditions. TEM micrographs were acquired using a JEOL JEM‐2100 microscope operated at 200 kV. Particle size distributions were determined by analyzing at least 300 nanoparticles using ImageJ software.^[^ ^87^ ^]^

Surface topography was analyzed in tapping mode using a Bruker Dimension Icon AFM. Images (1 × 1 µm^2^ to 10 × 10 µm^2^) were recorded in air.

ATR‐FTIR spectra were collected using a PerkinElmer Spectrum Two spectrometer with 1 cm^−1^ resolution in the range 4000–400 cm^−1^. Background spectra (air) were acquired before each measurement. Data were processed with Spectrum10 software and further baseline‐corrected in Origin2021.

### MTT Assay Toward Caco‐2 Cells

The human colon adenocarcinoma cell line Caco‐2 was purchased from ATCC (Rockville, MD, USA). Cells were maintained in Dulbecco's Modified Eagle Medium (DMEM; high glucose, with L‐glutamine) supplemented with 10% fetal bovine serum (FBS), 100 U mL^−1^ penicillin, and 100 µg mL^−1^ streptomycin. Cultures were kept at 37 °C in a humidified incubator with 5% CO_2_. For experimental assays, Caco‐2 cells were seeded into 48‐well plates at a density of 2 × 10⁵ cells per well and cultured overnight to allow proper adhesion. Cell viability was assessed using the MTT assay with 3‐(4,5‐dimethylthiazol‐2‐yl)‐2,5‐diphenyltetrazolium bromide (Sigma–Aldrich). Cells were then incubated with different types of hydrogels, cut into circular pieces with a diameter of 1 cm to ensure uniform quantities were applied to each well. Treatments were then added in triplicate wells for each condition. After 24 of exposure, the culture medium was removed, and 150 µL of MTT‐containing medium (0.5 mg mL^−1^) was added to each well. The cells were incubated at 37 °C for 2 h, after which the medium was discarded, and the resulting formazan crystals were dissolved in 150 µL of SDS‐DMF buffer (pH 4.7). Samples were then incubated at room temperature on an orbital shaker, protected from light, for 40 min. Absorbance was measured at 550 nm using a microplate reader (Glomax® Discover, Promega, Madison, WI, USA). All data were reported as the mean ± SD (*n* = 3). For comparisons of different groups, statistical significance was determined using one‐way ANOVA analysis. The software GraphPad, InStat3 (GraphPad Software, Inc.) was used for statistical analysis and *p*‐values < 0.05 were considered statistically significant.

## Conflict of Interest

Author Patrizia Nadia Hanieh was employed by the company Nanofaber Srl. The remaining authors declare that this research was conducted in the absence of any commercial or financial relationships that could be construed as a potential conflict of interest.

## Author Contributions

C.P. performed the measurements. V.M. co‐supervised C.P. during experimental work and performed data analysis. A.T. performed ATR‐FTIR measurements. A.B, and L.G. performed rheological, XRD and DMA measurements and analyzed rheological, XRD and DMA data. P.N.H., N.F. and A.R. performed SEM, AFM and biological studies. E.M., P.B. and L.T. supervised V.M. on data collecting and analysis. L.T., E.M., and P.B. conceived the experimental work, revised the manuscript and are responsible for funding acquisition. The final version was approved by all authors.

## Supporting information



Supporting Information

## Data Availability

The data that support the findings of this study are available from the corresponding author upon reasonable request.
