# Supplementary material for: Mussel‐Bioinspired Edible Ca2+‐Crosslinked Alginate Hydrogel Electrodes for Glucose Gastrointestinal Monitoring
Source: Adv Sci (Weinh). 2025 Dec 2;13(8):e16912. doi: 10.1002/advs.202516912 (PMC12884762; doi:10.1002/advs.202516912)
Supplement: Supplementary file 1 — Supporting Information [file ADVS-13-e16912-s001.docx]

Supporting Information

Mussel-bioinspired Edible Ca^2+^-crosslinked Alginate Hydrogel Electrodes for Glucose Gastrointestinal Monitoring

*Verdiana Marchianò,^1,2^ Claudio Pellegrini,^1^ Angelo Tricase,^2,3^ Eleonora Macchia,^2,3,4^ Andrea Brattelli,^5^ Luigi Gentile,^1,2^ Patrizia Nadia Hanieh,^6^ Noemi Fiaschini,^7^ Antonio Rinaldi,^8^ Luisa Torsi,^1,2,^* Paolo Bollella^1,2,^**

V. Marchianò, C. Pellegrini, L. Gentile, L. Torsi,* P. Bollella*

^1^Department of Chemistry, University of Bari Aldo Moro, Via E. Orabona 4, 70125 – Bari, Italy

^2^Centre for Colloid and Surface Science (CSGI@UniBa), University of Bari Aldo Moro, Via E. Orabona 4, 70125 – Bari, Italy

*E-mail corresponding Authors: [luisa.torsi@uniba.it](mailto:luisa.torsi@uniba.it), [paolo.bollella@uniba.it](mailto:paolo.bollella@uniba.it)

A. Tricase, E. Macchia
^2^Centre for Colloid and Surface Science (CSGI@UniBa), University of Bari Aldo Moro, Via E. Orabona 4, 70125 – Bari, Italy

^3^Department of Pharmacy-Pharmaceutical Sciences, University of Bari Aldo Moro, Via E. Orabona 4, 70125 – Bari, Italy

E. Macchia

^4^Faculty of Science and Engineering, Åbo Akademi University, 20500 Turku, Finland

A. Brattelli

^5^Aerospace Sciences and Engineering (Inter-University Ph.D.) Polytechnic of Bari, University of Bari Aldo Moro, Via Orabona 4, 70126 Bari, Italy

P. N. Hanieh,

^6^Nanofaber S.r.l., Via Anguillarese 301, 00123 Rome, Italy

N. Fiaschini

^7^Department of Sustainability, Circularity, and Climate Change Adaptation of Production and Territorial Systems (SSPT), Research Centre of Casaccia, ENEA, Via Anguillarese 301, Santa Maria di Galeria 00123, Rome, Italy.

A. Rinaldi

^8^Department of Energy Technologies and Renewable Sources (TERIN), Research Centre of Casaccia, ENEA, Via Anguillarese 301, Santa Maria di Galeria 00123, Rome, Italy.

***Table S1.*** *Experimental parameters obtained from equivalent circuit fitting of Nyquist plots derived Figures 1G and 1J for Ca^2+^-crosslinked alginate electrodes at 3 and 3.5% of alginate.*

| 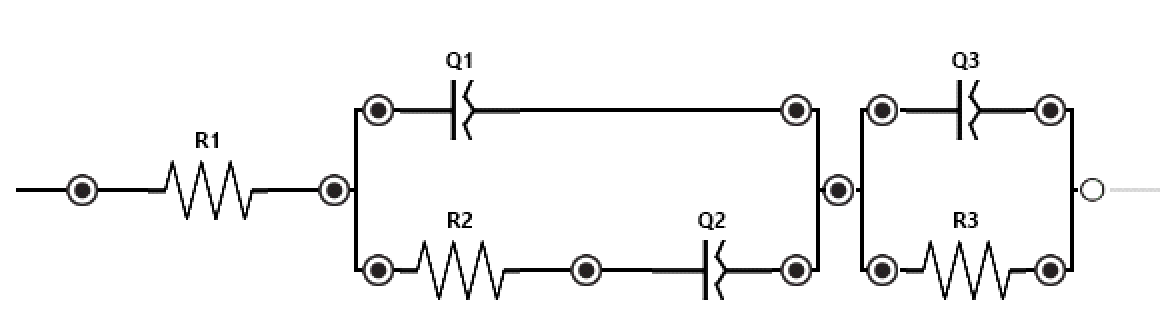 | | | | | | | | | | |
| --- | --- | --- | --- | --- | --- | --- | --- | --- | --- | --- |
|  | **R_S_ / Ω** | **CPE_1_ / µF** | **n** | **R_CT_ / Ω** | **CPE_2_ / µF** | **n** | **CPE_3_ / µF** | **n** | **R_CT2_ / Ω** | **χ^2^** |
| **Alg 3%** | 1597 ± 64 | 38 ± 2 | 0.8 ± 0.1 | 22750 ± 2958 | 49 ± 2 | 1 ± 0.1 | 186 ± 19 | 0.9 ± 0.1 | 2618 ± 212 | 0.0002 |
| 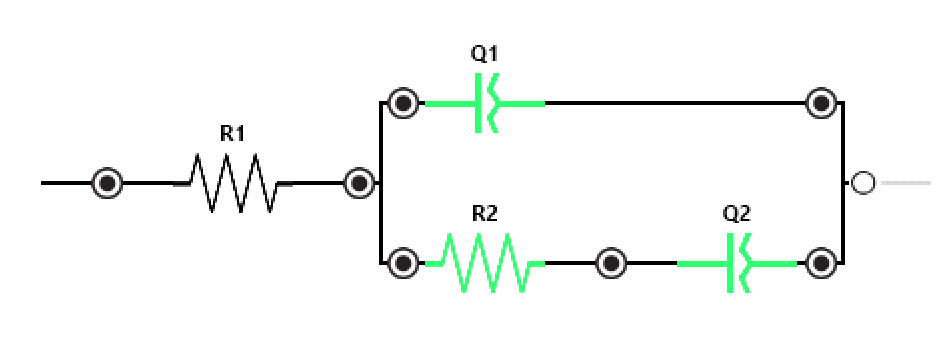 | | | | | | | | | | |
|  | **R_S_ / Ω** | **CPE_1_ / µF** | **n** | **R_CT_ / Ω** | **CPE_2_ / µF** | **n** |  |  |  | **χ^2^** |
| **Alg 3.5%** | 2270 ± 74 | 41 ± 1 | 0.9 ± 0.1 | 3779 ± 261 | 7 ± 1 | 0.8 ± 0.1 |  |  |  | 0.0014 |

***Table S2.*** *Experimental parameters obtained from equivalent circuit fitting of Nyquist plots derived Figures 2D, 2E and 2F for Ca^2+^-crosslinked alginate electrodes at 0% w/v, 2.5% w/v and 5% w/v glycerol.*

| **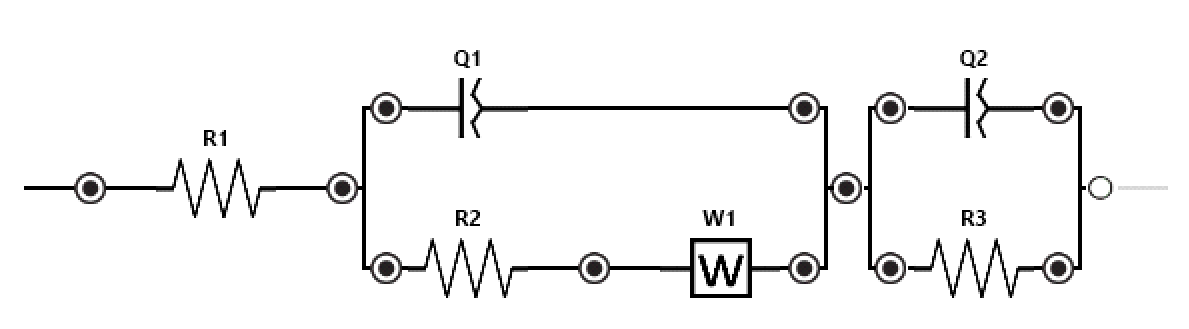** | | | | | | | | | |
| --- | --- | --- | --- | --- | --- | --- | --- | --- | --- |
|  | **R_S_ / Ω** | **CPE_1_ / µF** | **n** | **R_CT_ / Ω** | **Z_W_ / Ω** | **CPE_2_ / µF** | **n** | **R_CT2_ / Ω** | **χ^2^** |
| **Gly 0%** | 1074 ± 16 | 45 ± 1 | 1 ± 0.1 | 13210 ± 1334 | 3821 ± 385 | 75 ± 4 | 0.8 ± 0.1 | 2639 ± 405 | 0.0004 |
| **Gly 2.5%** | 284 ± 7 | 25 ± 2 | 0.9 ± 0.1 | 26200 ± 2442 | 8921 ± 624 | 8 ± 1 | 0.8 ± 0.1 | 1487 ± 97 | 0.0013 |
| **Gly 5%** | 826 ± 5 | 40 ± 2 | 1 ± 0.1 | 7730 ± 649 | 4916 ± 161 | 44 ± 4 | 0.8 ± 0.1 | 955 ± 87 | 0.0001 |

***Table S3.*** *Experimental parameters obtained from equivalent circuit fitting of Nyquist plots derived Figures 3D, 3E and 3F for Ca^2+^-crosslinked alginate electrodes at 0.5, 1 and 4 mL of polydopamine.*

| 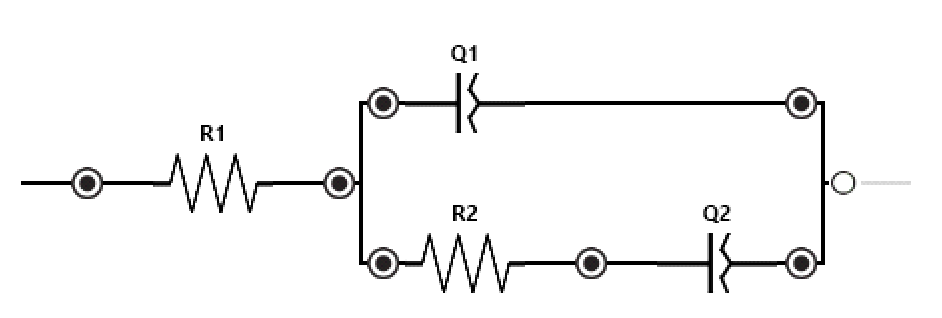 | | | | | | | | | | |
| --- | --- | --- | --- | --- | --- | --- | --- | --- | --- | --- |
|  | **R_S_ / Ω** | **CPE_1_ / µF** | **n** | **R_CT_ / Ω** | **CPE_2_ / µF** | **n** |  |  |  | **χ^2^** |
| **pDa 0.5 mL** | 2628 ± 73 | 5 ± 1 | 0.8 ± 0.1 | 1477 ± 99 | 15 ± 1 | 1 ± 0.1 |  |  |  | 0.0006 |
| 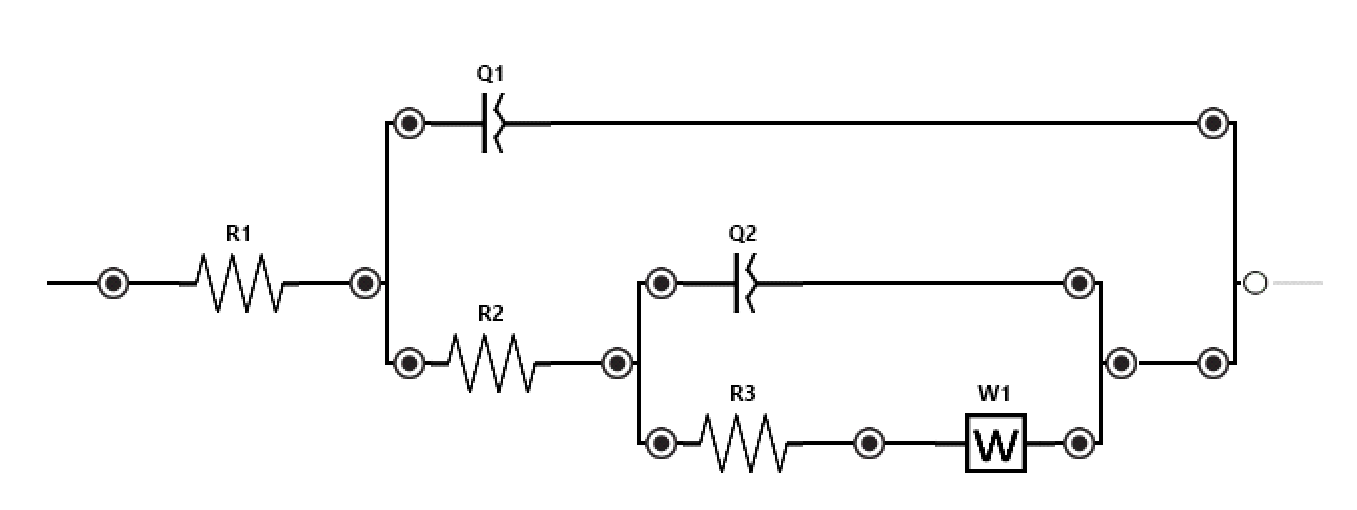 | | | | | | | | | | |
|  | **R_S_ / Ω** | **CPE_1_ / µF** | **n** | **R_CT_ / Ω** | **CPE_2_ / µF** | **n** | **R_CT2_ / Ω** | **Z_W_ / Ω** |  | **χ^2^** |
| **pDa 1 mL** | 1455 ± 7 | 19 ± 3 | 0.8 ± 0.1 | 1193 ± 75 | 29 ± 2 | 0.8 ± 0.1 | 7453 ± 375 | 5811 ± 168 |  | 4.7 x 10^-5^ |
| **pDa 4 mL** | 783 ± 6 | 25 ± 6 | 0.9 ± 0.1 | 1182 ± 30 | 43 ± 2 | 1 ± 0.1 | 5889 ± 243 | 4781 ± 130 |  | 4.1 x 10^-5^ |

***Table S4.*** *Kinetics and analytical parameters for GOx/AgNPs/pDA/Ca^2+^-crosslinked alginate hydrogel electrode*

| **GOx/AgNPs/pDA/Ca^2+^-crosslinked alginate hydrogel electrode** | |
| --- | --- |
| **K_m_^app^ / mM** | 0.35 ± 0.08 |
| ***I*_max_ / μA** | 13.8 ± 0.9 |
| **Sensitivity / μA mM^-1^** | 8.6 ± 0.6 |
| **LOD / µM** | 10.4 ± 0.8 |
| **Linear range / mM** | 0.05-1 |
| **Correlation Factor (R)** | 0.989 |
| **Applied Potential (V)** | -0.25 |

***Table S5.****Analytical performance comparison of glucose biosensors reported in recent literature (2025) and this work. Acronyms: pDA, polydopamine; AgNPs, silver nanoparticles; GOx, glucose oxidase; GrE, graphite electrode; GCE, glassy carbon electrode;* *PtCo, platinum-cobalt catalyst; PtNPs platinum nanoparticles; SPCE, screen-printed carbon electrode; n.r., not reported.*

| Electrode Platform | Applied Potential (vs Ref) | LoD | Linear Range | Sensitivity | Refs. |
| --- | --- | --- | --- | --- | --- |
| pDA–Ca²⁺–Alginate–AgNps–GOx | −0.25 V vs Ag/AgCl(sat) | 10.4 µM | 50 µM – 1 mM | 34.4 μA mM^−1^cm^−2^ | This Work |
| GrE/PtCo Nanozyme/GOx/Nafion | −0.30 V vs Ag/AgCl (3 M KCl) | 0.021 mM | 0.04 – 2.18 mM | 19.38 µA mM⁻¹ cm⁻² | [1] |
| GCE/PtNps + Chitosan/GOx | +0.65 V vs Ag/AgCl | 0.176 mM | 1 – 13 mM | 23.48 µA mM⁻¹ cm⁻² | [2] |
| SPCE Silk-Fibroin, GOx | +0.80 V vs Ag/AgCl | n.r. | 0.5 – 10 mM | 42.1 nA mm⁻² mM⁻¹ | [3] |

**

***Figure S1.*** *Bulk conductivity measurements of pristine pDA, pDA–Ca²⁺-crosslinked alginate, pDA–Ca²⁺-crosslinked alginate–AgNPs, and pDA–Ca²⁺-crosslinked alginate–AgNPs–GOx measured by linear four-point probe. Error bars indicate standard deviations from n = 3 independent measurements.*

**

***Figure S2.*** *Cumulative enzyme release over 6 h was quantified in simulated intestinal fluid (pH 6.8, 37 °C) for Ca^2+^-crosslinked alginate-GOx-FAM (red line) and pDA-Ca^2+^-crosslinked alginate-GOx-FAM (black line). Fluorescence intensity of FITC was recorded at λₑₓ = 485 ± 5 nm and λₑₘ = 520 ± 5 nm (excitation and emission slits= 5 nm).*


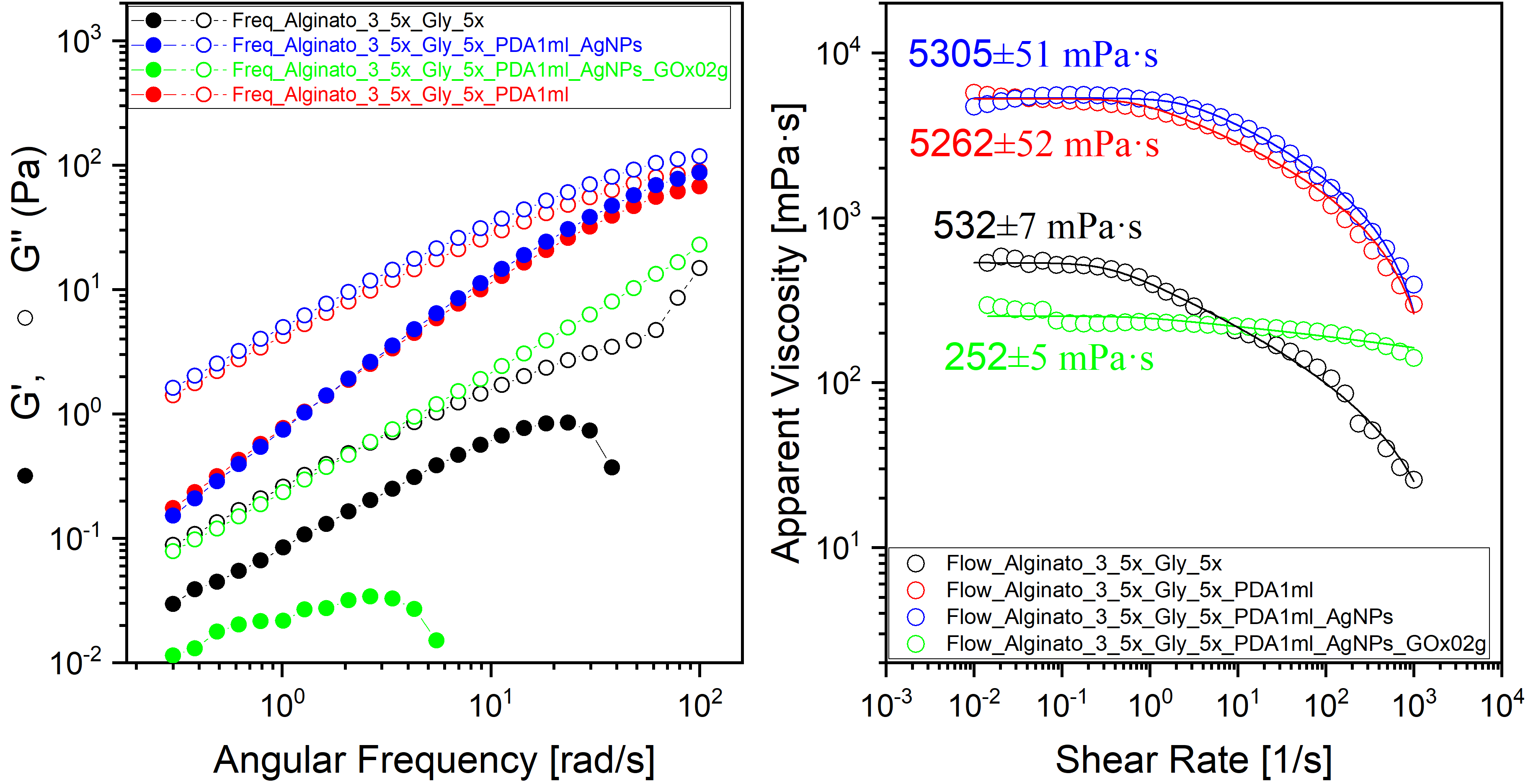


***Figure S3****. Frequency sweep* ***(A)*** *and flow curves* ***(B)*** *of the samples: alginate, pDA alginate, pDA alginate with AgNPs, pDA alginate with AgNPs and glucose oxidase.*


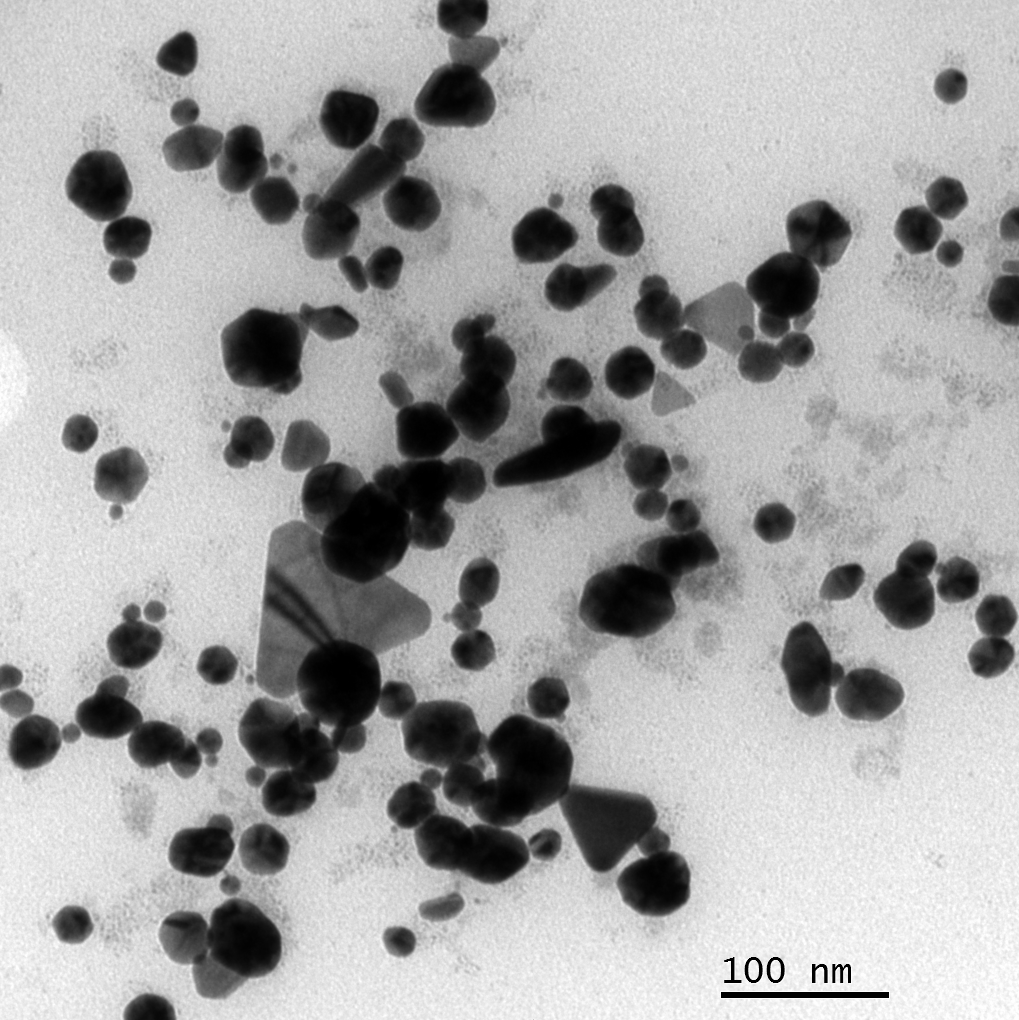


***Figure S4.*** *Transmission electron microscopy (TEM) image of silver nanoparticles (AgNPs) drop-cast on carbon-coated copper grids.*

***Figure S5.*** ***(A)****AFM image of sodium alginate-based hydrogel electrodes composed of 3.5% w/v sodium alginate crosslinked with calcium ions and containing 5% w/v glycerol;****(B)****AFM image of a soft electrode composed of 3.5% w/v sodium alginate crosslinked with calcium ions and containing 1 mL polydopamine and 5% w/v glycerol.*


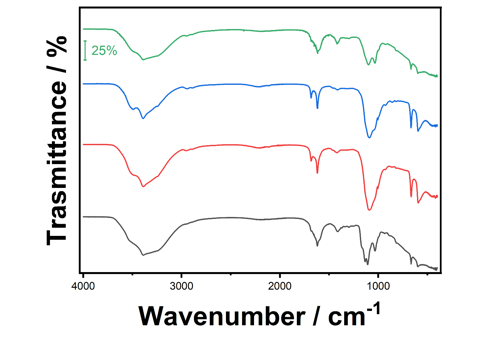


***Figure S6.*** *ATR-FTIR spectra of sodium alginate-based hydrogel electrodes (3.5% w/v) with 5% w/v glycerol in different formulations: base hydrogel (3.5% w/v sodium alginate + 5% w/v glycerol, green line), with the addition of 1 mL polydopamine (PDA, blue line), with 1 mL PDA and silver nanoparticles (AgNPs, red line), and with the further incorporation of 0.2 g glucose oxidase (GOx, black line).*

***Figure S7.*** *Caco-2 cell viability after exposure to hydrogel extracts (MTT, 24 h). Bars show normalized metabolic activity (%) for the positive control (Ctrl+), Ca²⁺–alginate (Ca²⁺–Alg), polydopamine-modified Ca²⁺–alginate (Ca²⁺–Alg–pDA), and AgNPs–containing pDA/alginate (Ca²⁺–Alg–pDA–AgNPs). All data are reported as the mean ± SD (n=3).*
